# Situational analysis of health systems for ear and hearing care in the World Health Organization (WHO) Eastern Mediterranean Region: A systematic review and evidence synthesis to inform national policies and strategies

**DOI:** 10.1016/j.ssmhs.2026.100170

**Published:** 2026-06

**Authors:** Dialechti Tsimpida, Hala Sakr, Abdelrahman Elwishahy, Shelly Chadha, Chander Chitra, Saied Mahmoudian

**Affiliations:** a Department of Gerontology, School of Economic, Social and Political Sciences, University of Southampton, Southampton, UK; b Department of Public Health, Policy and Systems, University of Liverpool, Liverpool, UK; c World Health Organization (WHO) Regional Office for the Eastern Mediterranean, Egypt; d Department of Noncommunicable Diseases, World Health Organization, Geneva, Switzerland; e Center for Ear and Nose Research, Head and Neck Research, Iran University of Medical Sciences Tehran, Iran

**Keywords:** Health systems, Ear and hearing care, Hearing loss, Health policy implementation, Universal health coverage, Eastern Mediterranean Region

## Abstract

In the Eastern Mediterranean Region (EMR), 78.1 million people experience hearing loss of any degree, with 22.1 million having disabling hearing loss, projected to reach 51.7 million by 2050. Unless global action is taken, the worldwide burden could reach over 700 million people with disabling hearing loss by 2050. This systematic review presents the first comprehensive health systems analysis of ear and hearing care (EHC) in the region. Following PRISMA guidelines, we analysed 146 articles through the WHO health systems framework to identify systemic barriers to effective EHC integration. Our findings reveal significant health systems challenges: fragmented governance with limited cross-sectoral coordination; inadequate financing with heavy reliance on out-of-pocket payments; critical workforce shortages across the region; and inequitable service distribution between urban and rural areas. While progress has been made with initiatives such as neonatal screening programs and primary care integration, these achievements remain limited in scope. Socioeconomic factors create additional barriers, affecting both hearing loss development and healthcare access. Alternative service delivery models, including telemedicine and task-sharing, show potential but lack systematic implementation. The economic burden of unaddressed hearing loss in the EMR ($30 billion annually) contrasts with potential returns of up to $7 per dollar invested. We propose five key actions: integrating EHC into universal health coverage, establishing comprehensive services across care levels, implementing awareness campaigns, developing monitoring systems, and promoting implementation research. This analysis provides evidence-based recommendations for health system reforms to address hearing loss while optimising resource allocation in diverse EMR contexts.

## Highlights

• The first comprehensive health systems analysis of Ear and Hearing Care in the Eastern Mediterranean Region.
• Consanguinity is a major contributor to hereditary hearing loss across the region.
• Five policy recommendations target gaps in governance, financing, workforce, and service delivery.
• Integrating EHC into universal health coverage could return $7 for every dollar invested.
• Task-sharing and telehealth can help optimise limited specialist workforces in diverse settings.

## Introduction

Hearing loss is a major public health issue and an important topic on the global health agenda (The Lancet Global Health, 2022, World Health Organization, 2021a). More than 1.5 billion people are living with some degree of hearing loss; that is a fifth of the global population. An estimated 430 million people have disabling hearing loss, i.e., a hearing threshold of over 35 dB HL in the better-hearing ear. Hearing loss is estimated to be the third largest cause of years lived with disability (YLD) and the leading cause of YLD for individuals older than 70 years (Haile et al., 2021). Furthermore, evidence has shown that even a mild loss of hearing may affect multiple aspects of an individual’s life if it remains unaddressed (Ferguson et al., 2017, Tsimpida et al., 2022a) or when individuals’ communication needs are unsupported (World Health Organization, 2021a, Haile et al., 2021).

Unless action is taken for hearing loss prevention and early intervention, WHO estimates that the numbers could rise to over 700 million people with moderate or greater hearing loss worldwide by 2050 (World Health Organization, 2021a), with the Eastern Mediterranean Region (EMR) projected to have the highest percentage increase (138.4 %) after the African Region (154.9 %) (Haile et al., 2021). The World Health Organization (WHO) EMR comprises 21 Member States and the occupied Palestinian territory (including East Jerusalem), covering a population of nearly 679 million people (World Health Organization,). Since an estimated 78.1 million people in the region live with hearing loss of any degree (World Health Organization, 2021a), hearing loss poses a substantial health burden in the EMR, a region already facing unique challenges in healthcare settings, fuelled by demographic changes, epidemiological transitions and multi-year conflicts. The EMR encompasses remarkable diversity in terms of socio-economic development, political stability, and healthcare infrastructure, ranging from high-income countries such as Saudi Arabia, Qatar, and the United Arab Emirates with well-established healthcare systems, to middle-income countries like Iran and Jordan with developing health infrastructures, and low-income or conflict-affected territories such as Palestine, Yemen, Somalia, and Syria, where healthcare delivery faces significant challenges. This heterogeneity creates varying capacities for implementing EHC services and necessitates context-specific approaches to health system strengthening (World Health Organization, 2021a). Despite this diversity, no previous study has provided a comprehensive, systematic analysis of EHC across the entire EMR, leaving policymakers without an evidence base for regional priority-setting and resource allocation. The region faces unique contextual challenges that distinguish it from other WHO regions, including high rates of consanguineous marriages (20–50 % across countries) that contribute significantly to genetic hearing loss, ongoing conflicts that disrupt healthcare delivery and create additional hearing health risks from blast injuries and trauma, rapid demographic transitions with varying age structures across countries, and extreme economic disparities that affect both hearing loss risk factors and access to care (World Health Organization, nd, World Health Organization, 2022). The *World report on hearing (*World Health Organization, 2021a*)*, published as a response to the World Health Assembly Resolution WHA70.13 in 2017 (World Health Organization, 2017a), recommends essential steps for scaling up the integration of people-centred EHC services into health plans at the country level. EHC requires the integration of a set of evidence-based interventions that include prevention, early diagnosis and treatment of ear and hearing problems (World Health Organization, 2017b). Compelling evidence shows that addressing the burden of hearing loss is feasible and cost-effective (The Lancet Global Health, 2022).

The integration of EHC into health systems represents a significant challenge that extends beyond clinical considerations to encompass broader social, economic, and organisational dimensions. Health systems in the EMR face unique contextual challenges, including fragmented governance structures, variable financing mechanisms, workforce shortages, and information system limitations. Approaching EHC through a health systems lens allows us to understand how these ear and hearing services intersect with all six building blocks of health systems as defined by WHO: leadership/governance, health care financing, health workforce, medical products and technologies, information and research, and service delivery. This social science perspective is particularly important in the EMR, where socioeconomic disparities, cultural factors, and conflict situations create complex barriers to equitable access and utilisation of EHC services. By conducting this situation analysis, we aim to not only assess clinical needs but also to understand the systemic and structural factors that influence how EHC can be effectively integrated and sustained within diverse health systems across the region.

According to the WHO framework for national health policies, strategies and plans (World Health Organization, 2010), a thorough situation analysis before health planning in countries is needed to develop an understanding of the planning context, available resources and capacities. This step is necessary to understand the epidemiology of hearing loss and the state of the systems supporting EHC that contain human resources and provide services (World Health Organization, 2015). Otherwise, the plans cannot be coherent, inclusive, and realistic because they may assume that capacities and resources are unavailable in the countries. Therefore, the current and projected demand for services would not be met (World Health Organization, 2010).

While several studies have been published in the countries of the EMR, to our knowledge, no previous study has been conducted to provide an overview of current resources in the WHO EMR towards a better understanding of the EHC landscape. Therefore, we conducted a systematic and comprehensive literature review to provide a situation analysis of EHC in the EMR.

Our systematic review was explicitly designed using the WHO health systems framework, thereby ensuring alignment with WHO recommendations and methodological consistency. The WHO Building Blocks framework serves as the current global standard for health systems analysis and offers several advantages for this situational analysis. First, it offers a consistent, standardised approach that enables systematic comparison across the diverse countries and territories of the EMR, ensuring comprehensive coverage of all essential health system components. Second, it aligns with the *World report on hearing* (World Health Organization, 2021a), which itself uses this framework, facilitating direct integration of our findings with global EHC policy guidance. Third, the framework has been validated and widely adopted across health systems strengthening initiatives globally, providing methodological rigour and credibility to our analysis.

What makes our contribution particularly valuable for the EMR is not the creation of entirely new analytical frameworks, but rather: (1) the first comprehensive, systematic synthesis of EHC evidence across all EMR countries using rigorous methodology; (2) the identification and quantification of region-specific barriers—including high rates of consanguineous marriages, ongoing conflicts affecting healthcare delivery, diverse economic conditions from high-income to conflict-affected settings, and unique demographic transitions; (3) the quantification of the economic burden and potential returns on investment specific to the EMR context; and (4) the contextualisation of evidence-based policy recommendations within the constraints and opportunities of diverse EMR health systems.

## Materials and methods

The systematic review followed a standardised methodology for searching, filtering, reviewing, critiquing, interpreting, synthesising and reporting findings (Pati and Lorusso, 2018). We followed the updated Preferred Reporting Items for Systematic Reviews and Meta-Analyses (PRISMA) guidelines statement to ensure methodological rigour and quality (Page et al., 2021) (Table 1 **in the Supplement**). The protocol for this review was registered at the International Prospective Register of Systematic Reviews (PROSPERO) (Systematic Review Registration: PROSPERO CRD42022368559).

**Table 1:** Included studies and their characteristics.

**No**	**Country**	**Bibliography**	**Key points for situational analysis**
1	Afghanistan	(Nasir et al., 2004)	Risk factors for hearing loss
	Bahrain	**-**	
	Djibouti	**-**	
2	Egypt	**(**Elshaer et al., 2023**)**	High noise exposure and hearing loss in a working population
3	Egypt	**(**Taha et al., 2023**)**	High noise exposure and hearing loss in a working population
4	Egypt	**(**Gibriel et al., 2019**)**	Genetic factors of non-syndromic hearing loss
5	Egypt	**(**Khairy et al., 2018**)**	Hearing loss in high-risk newborns
6	Egypt	**(**El-Badry et al., 2014**)**	Neuropathy as an associated risk factor for hearing loss in children
7	Egypt	**(**Yamamah et al., 2012**)**	Ear and hearing disorders of schoolchildren
8	Egypt	**(**Taha et al., 2010**)**	Prevalence and risk factors of hearing loss
9	Egypt	**(**Sanyelbhaa Talaat et al., 2009**)**	Prevalence of auditory neuropathy among infants and young children
10	Egypt	**(**Abdel-Hamid et al., 2007**)**	Prevalence and pattern of hearing loss at the national level
11	Iran (Islamic Republic of)	**(**Mohseni et al., 2025**)**	Genetic factors of hearing loss
12	Iran (Islamic Republic of)	**(**Esmaeili et al., 2025**)**	High noise exposure and hearing loss in a working population
13	Iran (Islamic Republic of)	**(**Nadri et al., 2024**)**	High noise exposure and hearing loss in a working population
14	Iran (Islamic Republic of)	**(**Ghosn et al., 2024**)**	Prevalence and risk factors of hearing loss
15	Iran (Islamic Republic of)	**(**Asghari et al., 2024**)**	High noise exposure and hearing loss in a working population
16	Iran (Islamic Republic of)	**(**Sudani et al., 2024**)**	Consanguinity and hereditary hearing loss
17	Iran (Islamic Republic of)	**(**Aliazami et al., 2023**)**	Genetic factors of hearing loss
18	Iran (Islamic Republic of)	**(**Etemadinezhad et al., 2023**)**	Risk factors of hearing loss
19	Iran (Islamic Republic of)	**(**Jafarzadeh et al., 2023**)**	Newborn hearing screening
20	Iran (Islamic Republic of)	**(**Mohammadi et al., 2023**)**	The effect of smoking as a risk factors of hearing loss
21	Iran (Islamic Republic of)	**(**Rahimi et al., 2023**)**	Socioeconomic inequalities as causes of hearing loss
22	Iran (Islamic Republic of)	**(**Vallian Broojeni et al., 2023**)**	Genetic factors of hearing loss
23	Iran (Islamic Republic of)	**(**Afshar et al., 2022**)**	Risk factors for severe and profound hearing loss in children
24	Iran (Islamic Republic of)	**(**Babanejad et al., 2022**)**	Genetic aetiology of hearing loss in Iran
25	Iran (Islamic Republic of)	**(**Gharibi and Khavidaki, 2022**)**	Newborn hearing screening
26	Iran (Islamic Republic of)	**(**Golbabaei Pasandi et al., 2022**)**	Risk factors of hearing loss
27	Iran (Islamic Republic of)	**(**Saraei et al., 2022**)**	Risk factors of hearing loss
28	Iran (Islamic Republic of)	**(**Mahmoudian et al., 2021**)**	Ear and hearing care program in Islamic Republic of Iran
29	Iran (Islamic Republic of)	**(**Moradi et al., 2021**)**	Rehabilitation of children with cochlear implant
30	Iran (Islamic Republic of)	**(**Jalali et al., 2020**)**	Prevalence of hearing loss among school-age children
31	Iran (Islamic Republic of)	**(**Koohiyan, 2020**)**	Genetics of hereditary hearing loss
32	Iran (Islamic Republic of)	**(**Hajilari et al., 2019**)**	Hereditary Hearing Loss and Consanguinity
33	Iran (Islamic Republic of)	**(**koohiyan, 2019**)**	Genetic factors of hearing loss in the Iranian population
34	Iran (Islamic Republic of)	**(**Monshizadeh et al., 2019**)**	Language intervention for children with cochlear implants
35	Iran (Islamic Republic of)	**(**Keihanidost et al., 2018**)**	Risk factors for hearing loss and its prevalence in neonates
36	Iran (Islamic Republic of)	**(**Saffari et al., 2018**)**	Prevalence of sensorineural hearing loss
37	Iran (Islamic Republic of)	**(**Zahed Pasha et al., 2018**)**	Screening of hearing in newborn infants
38	Iran (Islamic Republic of)	**(**Asghari et al., 2017**)**	Prevalence of hearing impairment by age and gender
39	Iran (Islamic Republic of)	**(**Ghasemnejad et al., 2017**)**	Non-syndromic hearing loss genes
40	Iran (Islamic Republic of)	**(**Saki et al., 2017**)**	Universal newborn screening and prevalence of deaf children
41	Iran (Islamic Republic of)	**(**Beheshtian et al., 2016**)**	Heterogeneity of hereditary hearing loss in Iran
42	Iran (Islamic Republic of)	**(**Tajik and Ahmadpour-kacho, 2016**)**	Early diagnosis and intervention for hearing loss in newborns
43	Iran (Islamic Republic of)	**(**Alaee et al., 2015**)**	Risk factors for hearing loss among high-risk infants
44	Iran (Islamic Republic of)	**(**Daneshi et al., 2015**)**	Paediatric cochlear implantation
45	Iran (Islamic Republic of)	**(**Farhat et al., 2015**)**	Hearing impairment among healthy and intensive care unit neonates
46	Iran (Islamic Republic of)	**(**Firoozbakht et al., 2014**)**	Community-based hearing screening program
47	Iran (Islamic Republic of)	**(**Haghshenas et al., 2014**)**	Auditory screening in infants
48	Iran (Islamic Republic of)	**(**Jeddi et al., 2014**)**	Aural rehabilitation in children with cochlear implants
49	Iran (Islamic Republic of)	**(**Panahi et al., 2014**)**	Thresholds in children with a history of neonatal hyperbilirubinemia
50	Iran (Islamic Republic of)	**(**Baradaranfar et al., 2011**)**	Hearing status in neonatal hyperbilirubinemia
51	Iran (Islamic Republic of)	**(**Mohammadzadeh et al., 2011**)**	Hearing outcomes in primary school children
52	Iran (Islamic Republic of)	**(**Mahdieh et al., 2010**)**	Genetic factors of non-syndromic hearing loss
53	Iran (Islamic Republic of)	**(**Sarafraz and Ahmadi, 2009**)**	Screening model for school-aged children
54	Iran (Islamic Republic of)	**(**Jafari et al., 2007**)**	Diagnosis, amplification, and intervention in deaf children
55	Iran (Islamic Republic of)	**(**Lotfi and Mehrkian, 2007**)**	Prevalence of auditory neuropathy in students
56	Iran (Islamic Republic of)	**(**Pouryaghoub et al., 2007**)**	Interaction of smoking and occupational noise exposure on hearing loss
57	Iraq	**(**Al Samarrai et al., 2024**)**	Risk factors of hearing loss
58	Iraq	**(**Al-Obeidy et al., 2019**)**	School-entry screening program for ear and hearing problems
59	Jordan	**(**Obeidat et al., 2024**)**	Newborn hearing screening
60	Jordan	(Almaayeh et al., 2018)	Prevalence of noise induced hearing loss among industrial workers
61	Jordan	**(**Al-Dababneh et al., 2016**)**	Educational needs of deaf and hard of hearing children
62	Jordan	**(**Abu-Shaheen et al., 2014**)**	Prevalence and risk factors of hearing loss among infants
63	Jordan	**(**Attias et al., 2006**)**	Prevalence of congenital and early-onset hearing loss
64	Jordan	**(**Medlej-Hashim et al., 2002**)**	Genetic factors of hearing loss
65	Kuwait	**(**Al-Kandari and Alshuaib, 2010**)**	Hearing evaluation of school children
66	Kuwait	**(**Al-Kandari and Alshuaib, 2007**)**	Newborn hearing screening
67	Lebanon	**(**Fooladi, 2012**)**	Effects of environmental noise on hearing health
68	Lebanon	**(**El Zir et al., 2008**)**	Environmental noise, smoking and age as combined risk factors
69	Lebanon	**(**Tabchi et al., 2000**)**	Epidemiology of profound neurosensory deafness in children
	Libya	**-**	
70	Morocco	**(**El Fizazi et al., 2024**)**	Genetic factors of hearing loss
71	Morocco	**(**AitRaise et al., 2023**)**	Genetic factors of hearing loss
72	Oman	**(**Khandekar et al., 2010**)**	Prevalence and determinants of hearing loss
73	Oman	**(**Khabori and Patton, 2008**)**	Consanguinity and deafness in children
74	Oman	**(**Khabori and Khandekar, 2007**)**	Prevalence of unilateral hearing loss
75	Oman	**(**Khandekar et al., 2006**)**	Neonatal screening for hearing loss
76	Oman	**(**Al Khabori and Khandekar, 2004**)**	Prevalence and risk factors of hearing loss
77	Oman	**(**Al Khabori, 2004**)**	Causes of severe to profound deafness in the paediatric population
78	Pakistan	**(**Shadab et al., 2024**)**	Genetic factors of hearing loss
79	Pakistan	**(**Naz, 2022**)**	Molecular genetic landscape of hereditary hearing loss in Pakistan.
80	Pakistan	**(**Ahmed et al., 2020**)**	Prevalence and features of inner ear malformations among children
81	Pakistan	**(**Doll et al., 2020**)**	Genetic spectrum of syndromic and non-syndromic hearing loss
82	Pakistan	**(**Zhou et al., 2020**)**	Mutations as factors of hearing loss in consanguineous Pakistani families.
83	Pakistan	**(**Mumtaz and Saqulain, 2020**)**	National neonatal hearing screening in Pakistan
84	Pakistan	**(**Mumtaz et al., 2019**)**	Neonatal hearing screening
85	Pakistan	**(**Richard et al., 2019**)**	Genetic factors contributed by consanguineous Pakistani families
86	Pakistan	**(**Ahmed et al., 2018**)**	Genetic factors, prevalence and screening for hearing loss in neonates
87	Pakistan	**(**Wasim et al., 2018**)**	Communication using Pakistan Sign Language (PSN)
88	Pakistan	**(**Qureshi et al., 2017**)**	Effects of bomb blast injury on the ears
89	Pakistan	**(**Mustafa et al., 2017**)**	Otitis media in children aged up to 5years
90	Pakistan	**(**Naz et al., 2017**)**	Genetic factors of moderate to severe hearing loss
91	Pakistan	**(**Shakoor et al., 2016**)**	Bacterial aetiology of otitis media in children
92	Pakistan	**(**Halim and Abbas, 2015**)**	Sign-language system for individuals with hearing loss
93	Pakistan	**(**Salman et al., 2015**)**	Genetic factors of moderate to severe hearing loss
94	Pakistan	**(**Yan et al., 2015**)**	Aetiology of congenital hearing loss
95	Pakistan	**(**Ibrahim and Bhutta, 2013**)**	Prevalence of hearing loss among children
96	Pakistan	**(**Raza et al., 2012**)**	Risk factors of hearing loss in children
97	Pakistan	**(**Musani et al., 2011**)**	Frequency and factors of hearing loss
98	Pakistan	**(**Sajjad et al., 2008**)**	Causes of childhood deafness and the role of consanguinity.
99	Pakistan	**(**Khan et al., 2007**)**	Cochlear implant programme for children and adults
100	Pakistan	**(**O’Hara et al., 2002**)**	Prevalence of hearing loss in siblings of deaf children
101	Palestine	**(**Shehabi et al., 2023**)**	High noise exposure and hearing loss in a working population
102	Palestine	**(**Corradin et al., 2014**)**	Infant hearing loss at a hospital in Bethlehem-Palestine
103	Qatar	**(**Alkhidir et al., 2024**)**	Prevalence and genetic factors of hearing loss
104	Qatar	**(**Girotto et al., 2014**)**	Consanguinity and hereditary hearing loss
105	Qatar	**(**Bener et al., 2005**)**	Prevalence and risk factors of hearing loss in infants
106	Saudi Arabia	**(**Alnoury et al., 2025**)**	High noise exposure and hearing loss in a working population
107	Saudi Arabia	**(**Aljabri et al., 2025**)**	Consanguinity and hereditary hearing loss
108	Saudi Arabia	**(**Al-Shaikh Sulaiman, 2024**)**	Newborn hearing screening
109	Saudi Arabia	**(**Alanazi et al., 2024**)**	Pre-school hearing screening
110	Saudi Arabia	**(**Almalki, 2024**)**	Prevalence and genetic factors of hearing loss
111	Saudi Arabia	**(**Alothman et al., 2024**)**	Universal newborn hearing screening
112	Saudi Arabia	**(**Alateeq et al., 2023**)**	Risk factors of hearing loss
113	Saudi Arabia	**(**Alzahrani et al., 2023**)**	Risk factors of hearing loss
114	Saudi Arabia	**(**ALqarny et al., 2021**)**	Correlations of hearing loss among adolescents, adults, and elderly
115	Saudi Arabia	**(**Alasim, 2020**)**	Inclusion programmes for deaf or hard-of-hearing students
116	Saudi Arabia	**(**Fageeh and Mansoor, 2020**)**	Guidance on health and hygiene practices in sign language
117	Saudi Arabia	**(**Alkahtani et al., 2019**)**	Identification and characteristics of sensorineural hearing loss in children
118	Saudi Arabia	**(**Halawani et al., 2019**)**	Post-operative care for cochlear implantation
119	Saudi Arabia	**(**Alharbi and Ahmed, 2015**)**	Evaluation of hearing loss among kindergarten children
120	Saudi Arabia	**(**Al-Mazrou et al., 2014**)**	Antimicrobial susceptibility of acute otitis media in children
121	Saudi Arabia	**(**Al-Rowaily et al., 2012**)**	Prevalence and types of hearing loss among school-entrant children
122	Saudi Arabia	**(**Al-Muhaimeed et al., 2009**)**	Cochlear implantation at a University Hospital, in Saudi Arabia
123	Saudi Arabia	**(**Habib and Abdelgaffar, 2005**)**	Neonatal hearing screening
124	Saudi Arabia	**(**Ahmed et al., 2004**)**	High noise exposure and hearing loss in a working population
125	Saudi Arabia	**(**Maisoun and Zakzouk, 2003**)**	Hearing screening of neonates
126	Saudi Arabia	**(**Al-Abduljawad and Zakzouk, 2003**)**	Prevalence of sensorineural hearing loss among children
127	Saudi Arabia	**(**Fageeh, 2004**)**	Hearing loss in schools for deaf children
128	Saudi Arabia	**(**Daghistani et al., 2002**)**	Hearing loss in low-birth-weight children
129	Saudi Arabia	**(**Zakzouk et al., 2002**)**	Epidemiology of acute otitis media in children
130	Saudi Arabia	**(**Zakzouk and Hajjaj, 2002**)**	Epidemiology of chronic suppurative otitis media among children
131	Saudi Arabia	**(**Ahmed et al., 2001**)**	Occupational noise exposure and hearing loss of workers
	Somalia	**-**	
132	Sudan	**(**Alier et al., 2025**)**	Prevalence and risk factors of hearing loss
133	Sudan	**(**Ahmed et al., 2017**)**	Risk factors and management of hearing loss in children
134	Syrian Arab Republic	**(**Yücel et al., 2019**)**	Newborn hearing screening
135	Syrian Arab Republic	**(**Kaheel et al., 2018**)**	Genetic factors of hearing loss
136	Syrian Arab Republic	**(**Moassass et al., 2018**)**	Genetic factors of hearing loss
137	Tunisia	**(**Romdhane et al., 2014**)**	Aspects of consanguinity: some examples from the Tunisian population
138	Tunisia	**(**Abed et al., 2013**)**	Hearing screening in newborns and infants
139	Tunisia	**(**Nouaili et al., 2010**)**	Newborn hearing screening
140	Tunisia	**(**Ben Arab et al., 2004**)**	Effect of consanguinity and endogamy in non-syndromic deafness
141	United Arab Emirates	**(**Elsayed and Al-Shamsi, 2022**)**	Genetic factors of hearing loss
142	United Arab Emirates	**(**Tlili et al., 2017**)**	Causes of hereditary hearing loss
143	United Arab Emirates	**(**Borders et al., 2016**)**	Newborn hearing screening and follow-up
144	United Arab Emirates	**(**Ur Rehman et al., 2012**)**	Hearing screening for neonates
145	Yemen	**(**Asaad et al., 2023**)**	Genetic factors of hearing loss
146	Yemen	**(**Al’shardzhabi and Tsygankova, 2014**)**	Prevalence of hearing loss among the elementary school pupils

## Selection and appraisal

### Search strategy and data sources

The search strategy included a combination of medical subject headings (MESH terms) and text words (provided as eMethods 1 in the Supplement). The following English language electronic bibliographic databases were searched for relevant studies published from 1 January 2000–10 May 2025: Cochrane Library, *ScienceDirect*, PubMed, Scopus, *Web of Science* and Embase. The searches were supplemented by hand searches of the reference lists of eligible studies.

### Inclusion criteria

Articles were included if they were reviews or original articles with observational design, with three concepts of interest:

1) *Population:* population residing *in* 21 Member States and the occupied Palestinian territory (including East Jerusalem),
2) *Issue of interest*: prevalence or incidence of hearing loss,
3) *Settings of interest*: ear and hearing care services.

Studies based in both clinical settings and population-level epidemiological studies were included if they met the inclusion criteria.

### Exclusion criteria

Articles written in languages other than English and published before January 2000 were excluded. The temporal restriction to post-2000 publications was chosen because this period coincides with when the World Health Assembly (WHA) endorsed a global strategy for the prevention and control of non-communicable diseases (World Health Organization, 2023a), marking a shift toward systematic approaches to chronic conditions including hearing loss. Additionally, healthcare delivery models, diagnostic technologies, and health system frameworks have evolved significantly since 2000, making earlier literature less relevant to current policy and practice contexts. Articles with study designs such as letters to editors, editorials, case reports, case series, randomised controlled trials, books, as well as studies without an available full text were also excluded.

### Selection of studies

The results of the systematic search were exported in “Zotero” app, to remove duplicate results. Study selection was completed in two stages. First, three reviewers independently screened the titles and abstracts of the retrieved articles and subsequently accessed and screened the full text of relevant studies against the eligibility criteria. Disagreements between reviewers were resolved through discussion and consensus. When consensus could not be reached, a third reviewer was consulted to make the final decision. The inter-rater reliability was calculated using Cohen's kappa coefficient (κ = 0.96).

### Data extraction and synthesis

The reviewers independently extracted data using an initial data extraction form piloted in five randomly selected studies. Descriptive information was extracted from the studies regarding publication characteristics (i.e., country of study, author(s), year of publication and key points for situational analysis). A data extraction table was developed, including the following elements of the selected studies: names of authors, publication year, country, and key point(s) for the situation analysis.

The final data extraction form included fields for methodological characteristics (study design, sample size, methods of hearing assessment), population characteristics (age groups, risk factors identified), and main outcomes related to EHC (prevalence data, service availability, intervention outcomes). The three reviewers who conducted the data extraction were trained in systematic review methodology and had backgrounds in public health, otolaryngology, and audiology, respectively.

The quality assessment (Thomas et al., 2004) of the included studies was done with reference to (i) selection bias, (ii) epidemiological study design, (iii) covariates and (iv) data collection methods. The studies were rated based on the following main criteria being met (maximum rating of 4): selection bias in terms of representativeness at the country level; epidemiological study design; included socio-economic covariates (e.g. education, occupation, income, wealth), given the steep social gradient in hearing health (Tsimpida et al., 2019); and reliable data collection methods (e.g. audiological examination). Each point given indicated the presence of the relevant criterion, and articles were rated as high (4 points), moderate (3 points), low (2 points) and very low (1 point or 0 points).

The data were synthesised based on situation analysis characteristics, as defined by the WHO framework for national health policies, strategies and plans (World Health Organization, 2010):

a) assessment of social determinants of health and health needs, including current and projected disease burdens and health challenges,
b) assessment of health system performance and of performance gaps in responding to needs and expectations,
c) assessment of the capacity of the health sector to respond to current and future challenges,
d) assessment of service delivery models in the region.

Therefore, the findings were categorised and presented based on the four predefined themes listed above.

We synthesised all available data and presented them using tables, including the date of their last update when necessary.

It is worth noting that this analytical approach using the WHO framework enables systematic stocktaking of EHC resources and gaps, but does not extend to analysing the causes of identified gaps, the political and institutional processes that created them, or the specific implementation pathways to address them. Such analyses would require different methodologies, including policy analysis, political economy studies, and implementation research, which fall beyond the scope of this situational analysis.

### Ethics and dissemination

Ethical approval was not required, as this study did not directly involve human subjects.

## Results

### Search results

**Fig. 1:**
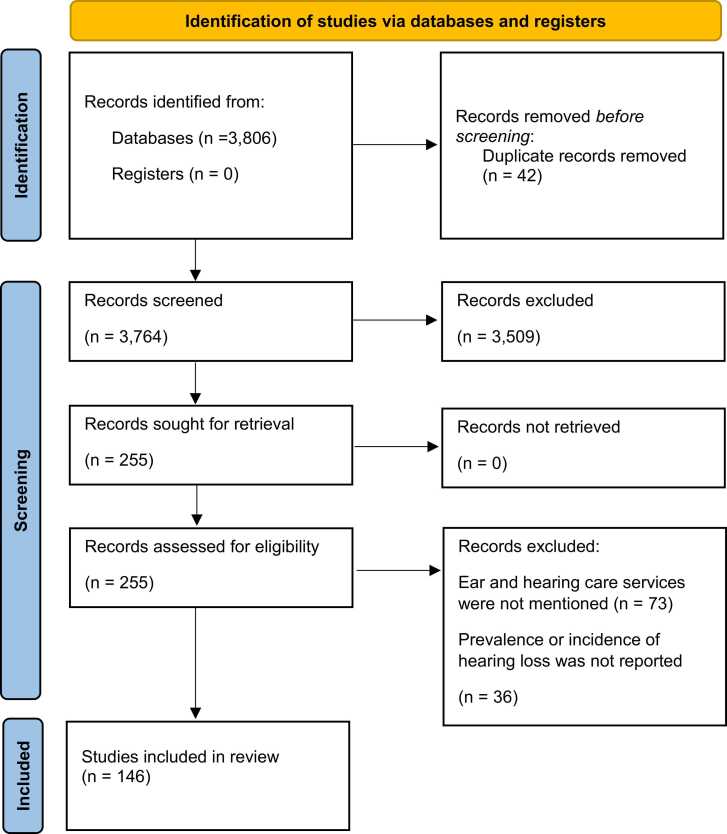
PRISMA 2020 flow diagram (Page et al., 2021).

The number of articles that were found in each sequential search step is shown in Fig. 1.

A total of 146 articles were included for critical review. The key points of these publications from countries in EMR include research on the genetic aetiology for hearing loss in children, evidence from community-based hearing screening programs, prevalence data and identified risk factors for hearing loss, as well as rehabilitation practices and language interventions. All included publications by country and the key points made in each study are listed in Table 1.

The results of quality appraisal of the included studies are reported in eTable 2 in the Supplement. The critical appraisal of the studies was based on their internal validity and not as a marker for the credibility of evidence in informing the situational analysis as per inclusion or exclusion criteria (Petticrew, 2003). Quality appraisal revealed considerable variation in study methodology and reporting. Of the 146 included studies, 7 were rated as high quality (4 points), 27 as moderate quality (3 points), 54 as low quality (2 points), and 31 as very low quality (1 point or 0 points). Seventeen studies were literature reviews and did not receive methodological ratings. The main quality limitations identified were selection bias, lack of representative sampling at country level, limited inclusion of socioeconomic covariates, and varying data collection methods across studies Below, we present the findings from the systematic review according to the four predefined themes of the WHO framework for national health policies, strategies and plans (World Health Organization, 2010):

a) *Assessment of social determinants of health and health needs, including current and projected disease burdens and health challenges*
  Regarding the hearing loss burden in the region, it is estimated that more than 78.1 million people currently live with hearing loss in the WHO’s EMR, which represents 11 % of the region’s total population (World Health Organization, 2021a) (Table 2). Of those, an estimated 22.1 million people (3.1 %) live with disabling hearing loss. Unless action is taken to prevent and address hearing loss in the EMR, the numbers could rise to over 194 million by 2050 (World Health Organization, 2021a, Haile et al., 2021). In addition, the cases of disabling hearing loss in the EMR are projected to increase from 22.1 million in 2020–29.7 million in 2030, 39.5 million in 2040, and 51.7 million in 2050, respectively (World Health Organization, 2021a). The estimated number and prevalence of hearing loss among age groups is shown in Table 3.

**Table 2:** Total number and prevalence of hearing loss in the sector in countries and territories of the WHO Eastern Mediterranean Region for all age groups according to severity.

**Total hearing loss (20 +dB)**	**78.1 million (10.97 %)**
**Disabling hearing loss (35 +dB)**	**22.12 million (3.11 %)**
Mild (20–34 dB)	56 million (7.86)
Moderate (35–49 dB)	13.27 million (1.86 %)
Moderately severe (50–64 dB)	5.30 million (0.74 %)
Severe (65–79 dB)	1.81 million (0.26 %)
Profound (80–94 dB)	1.08 million (0.15 %)
Complete (95 +dB)	0.65 million (0.09 %)

Data Source: World report on hearing (World Health Organization, 2021a)

**Table 3:** Total number and prevalence of hearing loss among age groups.

**Age groups**	**Total hearing loss**	**Disabling hearing loss**
0–5 years	1.30 million (1.54 %)	.45 million (0.52 %)
5 – 15 years	4.53 million (3.02 %)	1.58 million (1.05 %)
15 – 60 years	47.66 million (11.17 %)	10 million (2.35 %)
60 plus years	26.8 million (53.06 %)	10.4 million (19.28 %)

Data Source: World report on hearing (World Health Organization, 2021a)
  Consanguinity emerged as a particularly significant risk factor in the EMR region, with multiple studies documenting its role in hereditary hearing loss. The prevalence of consanguineous marriages ranges from 20 % to 50 % across different countries in the region, contributing substantially to the genetic causes of hearing loss among children. Overall, the major risk factors for hearing loss in the EMR, identified in this review, are consanguinity, genetic factors and otitis media, which are the leading causes of hearing loss among children (Nasir et al., 2004, Gibriel et al., 2019, El-Badry et al., 2014, Sanyelbhaa Talaat et al., 2009, Sudani et al., 2024, Aliazami et al., 2023, Vallian Broojeni et al., 2023, Afshar et al., 2022, Babanejad et al., 2022, Koohiyan, 2020, Hajilari et al., 2019, koohiyan, 2019, Ghasemnejad et al., 2017, Beheshtian et al., 2016, Alaee et al., 2015, Panahi et al., 2014, Baradaranfar et al., 2011, Mahdieh et al., 2010, Lotfi and Mehrkian, 2007, Attias et al., 2006, Medlej-Hashim et al., 2002, El Fizazi et al., 2024, AitRaise et al., 2023, Khabori and Patton, 2008, Al Khabori, 2004, Naz, 2022, Ahmed et al., 2020, Doll et al., 2020, Zhou et al., 2020, Richard et al., 2019, Ahmed et al., 2018, Mustafa et al., 2017, Naz et al., 2017, Shakoor et al., 2016, Salman et al., 2015, Yan et al., 2015, Sajjad et al., 2008, Alkhidir et al., 2024, Girotto et al., 2014, Almalki, 2024, Al-Mazrou et al., 2014, Zakzouk et al., 2002, Zakzouk and Hajjaj, 2002, Kaheel et al., 2018, Moassass et al., 2018, Romdhane et al., 2014, Ben Arab et al., 2004, Elsayed and Al-Shamsi, 2022, Tlili et al., 2017, Asaad et al., 2023, Shadab et al., 2024, Mohseni et al., 2025, Aljabri et al., 2025), and high or extended exposure to noise and loud sounds at work or leisure time (Elshaer et al., 2023, Taha et al., 2023, Asghari et al., 2024, Etemadinezhad et al., 2023, Golbabaei Pasandi et al., 2022, Almaayeh et al., 2018, Shehabi et al., 2023, Alzahrani et al., 2023, Ahmed et al., 2004, Ahmed et al., 2001, Nadri et al., 2024, Alnoury et al., 2025, Esmaeili et al., 2025), and from bomb blast injury (Qureshi et al., 2017). Also, the combined effect of noise with other risk factors, such as smoking (Mohammadi et al., 2023, Pouryaghoub et al., 2007, El Zir et al., 2008, Alateeq et al., 2023) and solvent exposure (Saraei et al., 2022), as well as dietary factors (Ghosn et al., 2024, Al Samarrai et al., 2024), were reported. Other factors include infectious diseases such as meningitis, measles, mumps, or rubella; and the use of ototoxic medications (World Health Organization, 2022). Socioeconomic factors linked to hearing loss include lower educational attainment, unemployment, and limited access to healthcare services (Alier et al., 2025). These factors can affect both hearing loss development and access to diagnosis and treatment, creating a cycle of disadvantage (Rahimi et al., 2023).
  The prevalence statistics of hearing loss are intricately linked to the population demographics within each country (World Health Organization, 2021a). Only a small number of studies in the situation analysis were reporting actual prevalence data (Taha et al., 2010, Abdel-Hamid et al., 2007, Jalali et al., 2020, Saffari et al., 2018, Asghari et al., 2017, Tabchi et al., 2000, Khandekar et al., 2010, Khabori and Khandekar, 2007, Al Khabori and Khandekar, 2004, Ibrahim and Bhutta, 2013, Musani et al., 2011, O’Hara et al., 2002, ALqarny et al., 2021, Alkahtani et al., 2019, Al-Abduljawad and Zakzouk, 2003). To provide a comprehensive overview, we have gathered available demographic data from the World Health Organization's regional office for the EMR. The Supplementary file includes **eTable 3**, which presents the population size (in thousands) of EMR countries, and **e**Table 4, which outlines the net primary school enrolment ratio per 100 school-age children residing in those countries.

**Table 4:** Population size and health sector capacity in countries and territories of the WHO Eastern Mediterranean Region in 2018.

**Country**	**Population size (in thousands)**	**Universal Health Coverage (UHC) service coverage index**^a^	**Primary health care facilities (per 10,000 population)**	**Physicians** **(per 10,000 population)**	**Pharmacists** **(per 10,000 population)**	**Nursing and midwifery** **(per 10,000 population)**	**Dentists** **(per 10,000 population)**
Afghanistan	31,575	37	0.8	4.0	0.7	3.2	0.4
Bahrain	1501	77	0.2	22.6	5.4	45.4	3.7
Djibouti	860	47	0.6	2.1	2.2	5.1	0.2
Egypt	92,115	68	0.6	8.2	4.9	15.5	2.1
Iran (Islamic Republic of)	79,926	72	3.5	15.4	3.0	21.3	4.0
Iraq	38,124	61	0.7	9.1	3.3	21.2	3.1
Jordan	10,309	76	7.0	23.0	13.1	33.2	7.2
Kuwait	4564	76	0.2	25.3	6.9	67.2	7.2
Lebanon	4485	73	0.5	31.2	19.8	37.4	15.6
Libya	6588	64	2.1	22.9	6.2	68.8	8.8
Morocco	35,220	70	0.8	6.7	2.6	8.5	1.1
Oman	4602	69	0.5	21.0	5.9	44.0	3.1
Pakistan	207,774	45	0.5	9.6	1.6	4.9	1.0
Palestine	4854	64	1.6	21.5	11.2	25.9	7.1
Qatar	2760	68	3.2	25.0	9.0	73.2	6.3
Saudi Arabia	33,414	74	0.7	26.4	8.7	55.2	5.0
Somalia	12,316	25	1.9	0.3	0.1	0.8	0.1
Sudan	41,985	44	1.5	2.8	2.4	33.5	2.1
Syrian Arab Republic	15,353	60	0.8	11.7	9.6	14.0	6.8
Tunisia	11,435	70	1.9	13.0	2.3	38.9	3.1
United Arab Emirates	9304	76	3.8	24.8	8.5	57.9	6.0
Yemen	28,170	42	1.4	1.7	1.0	6.3	0.2

a The indicator is an index reported on a unitless scale of 0–100, which is computed as the geometric mean of 14 tracer indicators of health service coverage (World Health Organization, 2019). Data source: WHO regional office of the Eastern Mediterranean (last update: 19 June, 2020)
  Recognising the coexistence of hearing loss with other health conditions (Tsimpida et al., 2021), we have deemed it helpful to also present the burden of well-documented diseases commonly associated with hearing loss, given that the latest is not well recorded. It is worth noting that within the EMR, the two leading causes of mortality were ischaemic disease and stroke (GBD, 2019, 2022), with individuals experiencing hearing loss being at a significantly higher risk of developing these conditions (Khosravipour and Rajati, 2021). eTable 5 in the Supplement provides a summary of the disease burden (measured as deaths per 100,000) in 2019 for both sexes and all age groups.
b) *Assessment of health system performance and of performance gaps in responding to needs and expectations*
  Several studies that have been included in the review have commended the estimated shortage of the EHC workforce, which is a common challenge in low-and-middle-income countries globally (Waterworth et al., 2022, Dillard et al., 2024). The most comprehensive evidence on the availability of the EHC workforce was published by Kamenov and colleagues (Kamenov et al., 2021). According to the available data on the number of EHC professionals per million population in EMR, five countries have between 11 and 50 ENT specialists per million, while another four report between 1 and 10 audiologists per million. Eight countries have five or fewer audiologists per million of the population, and only two countries have more than five audiologists per million. Six countries have five or fewer speech and language therapists/pathologists per million, and only two countries have more than five speech and language therapists/pathologists per million of the population. Four countries have five or fewer teachers of the deaf per million of the population, and only two countries have more than five teachers of the deaf per million of the population. Beyond the above aggregate regional data, detailed figures on the EHC workforce have been reported only in a study in the Islamic Republic of Iran, which identified 109 audiologists in the private sector and 135 speech and language therapists in the public sector (Mahmoudian et al., 2021).
  Financing mechanisms for EHC services vary considerably across the region, with significant implications for accessibility and sustainability. Our analysis found that only 7 out of 22 countries in the region have included hearing aids and related services in their universal health coverage packages or national health insurance schemes (Kamenov et al., 2021). This gap in financial protection means that in most EMR countries, EHC services—particularly hearing aids and rehabilitative services—rely heavily on out-of-pocket payments by users.
  The estimated annual cost of providing basic EHC services at the primary care level ranges from US$1–3 per capita in low-income countries to US$4–7 in middle-income countries of the region (World Health Organization, 2022, McDaid et al., 2021). However, current health system expenditure on EHC is significantly below these levels in most EMR countries, with many allocating less than US$0.50 per capita annually to these services (World Health Organization, 2022).
  Governance structures for EHC also show considerable fragmentation. In most EMR countries, responsibility for different aspects of EHC is divided across multiple ministries and departments, including health, education, social welfare, and labour. This fragmentation creates challenges for policy coherence, resource allocation, and continuity of care. Only three countries in the region (Saudi Arabia, Iran, and Oman) have established national committees or coordinating bodies specifically for EHC (World Health Organization, 2022, Al Khabori, 2004, Mahmoudian et al., 2021). Where such governance mechanisms exist, they have facilitated more integrated approaches to policy development and service delivery.
  The fragmented governance is particularly evident in how screening programs are implemented. While newborn hearing screening has been initiated in several countries, it often operates in isolation from other EHC services, creating challenges for referral pathways and continuum of care. This disconnection between detection and intervention represents a critical health system failure that must be addressed through more integrated governance approaches.
c) *Assessment of the capacity of the health sector to respond to current and future challenges*
  From the literature, it was apparent that there is a shortage of EHC services in the region, and the capacity to respond to current and future challenges is limited. However, according to the available evidence, the following countries have demonstrated some examples of EHC provision through their health systems: Djibouti and the Islamic Republic of Iran are advancing neonatal and early childhood screening for hearing loss (Mahmoudian et al., 2021); Egypt has expanded its EHC programme in primary healthcare units (Yamamah et al., 2012); Saudi Arabia has made neonatal hearing screening mandatory across the country, including in private clinics (World Health Organization, 2022). Assessment of newborn hearing screening programmes and their progress is also reported in studies from Iran (Jafarzadeh et al., 2023, Gharibi and Khavidaki, 2022, Keihanidost et al., 2018, Zahed Pasha et al., 2018, Firoozbakht et al., 2014), Jordan (Abu-Shaheen et al., 2014, Obeidat et al., 2024), Kuwait (Al-Kandari and Alshuaib, 2007), Oman (Khandekar et al., 2006), Pakistan (Mumtaz et al., 2019), Saudi Arabia (Maisoun and Zakzouk, 2003, Al-Shaikh Sulaiman, 2024), Tunisia (Abed et al., 2013) as well as screening models for preschool and school-aged children in Iran (Haghshenas et al., 2014, Mohammadzadeh et al., 2011, Sarafraz and Ahmadi, 2009), Iraq (Al-Obeidy et al., 2019), Kuwait (Al-Kandari and Alshuaib, 2010), Saudi Arabia (Alharbi and Ahmed, 2015, Al-Rowaily et al., 2012, Fageeh, 2004, Daghistani et al., 2002, Alanazi et al., 2024), and Yemen (Al’shardzhabi and Tsygankova, 2014).
  Regarding the capacity of the health sector in terms of primary care facilities and health professionals, the latest available data is shown in Table 4. Concerning hearing loss, it is important to note that over half of the countries in the EMR (13 out of 21) have achieved the desirable doctor-population ratio of 1:1000 (Kumar and Pal, 2018). This indicates that these countries have a relatively sufficient number of healthcare professionals who could, with the appropriate training, provide primary EHC alongside other primary care services.
  The study conducted by Kamenov and colleagues (Kamenov et al., 2021) presents successful examples of task sharing in the field of EHC. One such example is the training of 12,000 professional nurses in Zambia over a period of two weeks, enabling them to perform hearing tests. In India, science graduates receive a six-week training to fit and maintain hearing aids for individuals with moderate to severe hearing loss (Emerson et al., 2013). In New Delhi, general practitioners and primary healthcare workers have played a critical role in improving the identification of EHC issues within the community. By diagnosing acute or chronic otitis media in children, they have contributed to early intervention efforts (Bright et al., 2019, Suen et al., 2019). Similar positive practices have been observed in Djibouti, where training for doctors and midwives is underway to enable early childhood screening (World Health Organization, 2022).
  Examples of secondary and tertiary provision such as diagnosis, amplification and intervention such as rehabilitation with cochlear impacts were reported from studies conducted in Egypt (Khairy et al., 2018), Iran (Moradi et al., 2021, Monshizadeh et al., 2019, Tajik and Ahmadpour-kacho, 2016, Daneshi et al., 2015, Jeddi et al., 2014, Jafari et al., 2007), Pakistan (Raza et al., 2012, Khan et al., 2007), Palestine (Corradin et al., 2014), Saudi Arabia (Halawani et al., 2019, Al-Muhaimeed et al., 2009), Sudan (Ahmed et al., 2017).
d) *Assessment of service delivery models in the region*

Based on the published literature, several countries have established universal newborn screening (Mahmoudian et al., 2021, Jafarzadeh et al., 2023, Gharibi and Khavidaki, 2022, Saki et al., 2017, Farhat et al., 2015, Mumtaz and Saqulain, 2020, Alothman et al., 2024, Habib and Abdelgaffar, 2005, Yücel et al., 2019, Nouaili et al., 2010, Borders et al., 2016, Ur Rehman et al., 2012). However, more detailed data were retrieved by informant replies only for nine countries in EMR (Neumann et al., 2020); therefore, further country-specific situation assessments are needed (see eTable 6 in the Supplement).

Several countries demonstrated examples of alternative service delivery models to respond to current and future challenges, such as telemedicine/tele-audiology. For instance, virtual and remote EHC services were established due to the COVID-19 pandemic in several countries in the region (Zaitoun et al., 2022). Also, in Saudi Arabia, remote EHC services are being provided, such as remote programming of hearing aids (World Health Organization, 2022).

Regarding informational resources and the communication needs of people who communicate in sign language, Saudi Arabia has ensured that persons with hearing loss have access to media messages on all national broadcasting channels and also that adults with hearing loss can be provided with hygiene instructions in sign language (Fageeh and Mansoor, 2020). In addition, a mobile application was launched recently in Saudi Arabia, providing services to answer questions in sign language 24 h daily (Fageeh and Mansoor, 2020). Pakistan developed school curricula in sign language for all grades and online sign language education courses targeting the whole country.

In addition, a new software tool has been developed in Pakistan to detect sign language gestures and later translate the gesture into a spoken language to enable those who communicate in sign language to communicate with other members of society (Halim and Abbas, 2015, Wasim et al., 2018). Oman and the Islamic Republic of Iran have developed sign language courses for healthcare workers. Oman embarked on mass dissemination of educational videos during the COVID-19 pandemic, targeting people with disabilities, including those with hearing loss and their caregivers (World Health Organization, 2022). These educational videos featured sign language interpretation, closed captions, and simplified visual instructions to ensure accessibility for people with hearing loss and their caregivers. These good practices included teacher training in sign language, classroom acoustic modifications, peer integration programs, and specialized educational materials. Saudi Arabia has implemented inclusive education models where deaf and hard-of-hearing students learn alongside hearing peers with appropriate accommodations (Alasim, 2020). Also, a study in Jordan considered competencies that teachers need when teaching deaf or hard-of-hearing children (Al-Dababneh et al., 2016).

## Discussion

### Summary of main findings

The literature review included 146 studies in 21 Member States and the occupied Palestinian territory (including East Jerusalem) in the EMR and highlighted a range of characteristics of the current situation in EHC. It is currently estimated that more than 78.1 million people in the EMR live with hearing loss. The key findings from the reviewed publications from countries in the EMR are focused on the genetic causes of hearing loss in children, the impact of community-based hearing screening programs, the prevalence of hearing loss and related risk factors, and the available rehabilitation and language interventions. Main risk factors for hearing loss in the EMR as identified from the situation analysis include consanguinity, genetic factors, otitis media, prolonged exposure to loud noises at work or leisure, bomb blast injuries, smoking, solvent exposure, dietary factors, infectious diseases (e.g., meningitis, measles), and ototoxic medication use. Socioeconomic factors are also linked to hearing loss.

There is a shortage of EHC services and limited capacity to address current and future challenges in the EHC sector, as noted in several studies included in the review. While some countries in the EMR have implemented universal newborn screening, information for other age groups is limited. Despite the challenges in providing EHC, countries such as Djibouti, Iran, Egypt, and Saudi Arabia have made progress and have been identified as examples of good practices in EHC. However, the health sector in the region still faces significant performance gaps in addressing the needs and expectations of those with hearing loss. As a result, alternative service delivery models such as telemedicine and tele-audiology are emerging. No public health programs or interventions in the EMR have been evaluated for national implementation to raise awareness about hearing loss prevention throughout the lifespan.

In the next section, we discuss the findings and present suggested actions based on the situation analysis to improve the state of EHC in EMRO, addressing the challenges and implementing the recommendations of the *World Report on Hearing* (World Health Organization, 2021a).

a) *Assessment of social determinants of health and health needs, including current and projected disease burdens and health challenges*
  Regarding the hearing loss burden in the region, the present situation analysis showed that, to date, nationally representative studies for prevalence estimates and disease burden of hearing loss are lacking. The prevalence estimates presented have been based on age demographics and projections, not actual audiological data collected through hearing-related indicators in the national health information systems, which may vary geographically (Tsimpida et al., 2020, Tsimpida et al., 2022b). Since nationally representative epidemiological surveys on hearing loss are scarce (Pascolini and Smith, 2009), the lack of robust epidemiological data on hearing loss prevalence, including in this review, poses challenges in understanding the magnitude of the disease burden. It may be the case that hearing loss prevalence in EMR is more prevalent than currently reported (ALqarny et al., 2021).
  Understanding the magnitude of the problem and building an evidence-based rationale is necessary for scaling up governmental support and investment in EHC. Therefore, public health surveillance systems need to include hearing loss among the non-communicable diseases’ health indicators that are systematically collected, analysed and interpreted (Tsimpida et al., 2025). Regularly monitoring EHC needs will help track progress toward the goals specified in the *World report on hearing (*World Health Organization, 2021a*)*.
  Regarding the major risk factors for hearing loss, more research is needed to identify preventable risk factors of hearing loss due to environmental, occupational and recreational noise and lifestyle factors to address the risk of hearing loss in several population age groups in the EMR (World Health Organization, 2022, Fooladi, 2012). In addition, public health strategies are needed to improve hearing health literacy in the population living in the EMR (Shams et al., 2020, Wikkeling-Scott et al., 2019), increase awareness of the preventable risk factors and reduce individual and community-level stigma around diagnosis and treatment for hearing loss (McMahon et al., 2021).
  Despite the established effectiveness of hearing aids for addressing hearing loss, there is limited data on their utilisation in the EMR. Studies included in this review did not comprehensively report on hearing aid usage rates or barriers to amplification adoption. Potential barriers may include cost, limited awareness, social stigma, and insufficient fitting and follow-up services. Future EHC planning should prioritise increasing access to affordable hearing aids and necessary support services through strategies such as bulk purchasing, teleaudiology for remote fitting and adjustment, and training of primary care providers to support hearing aid users.
  Growing evidence indicates that hearing loss often co-occurs with cardiometabolic diseases, stroke, or diabetes, which are major health challenges in the EMR. Additionally, hearing loss can create substantial communication barriers in healthcare settings, potentially leading to delayed detection of other health issues and negatively impacting individuals' health-related quality of life (Tsimpida et al., 2021). However, it is worth noting that only one study examining the comorbidity of hearing loss with stroke has been identified in the EMR (Khosravipour and Rajati, 2021). This suggests the need for further research in this area to better understand the relationship between hearing loss and related comorbidities (Tsimpida et al., 2021). Such insights are crucial for identifying potential avenues for eliminating preventable cases of hearing loss, such as those related to diabetes.
b) *Assessment of health system performance and of performance gaps in responding to needs and expectations*
  The study published by Kamenov and colleagues (Kamenov et al., 2021) offers valuable insights into the capacity of the EMR to address present and future challenges. Given the significant gaps in this capacity and the inequalities within countries regarding the availability of EHC professionals and services, it is essential to gather detailed information on the number and distribution of these professionals (Waterworth et al., 2022). Access to such data is crucial for making informed policy decisions in each country or territory (Bhutta, 2019).
  The available data also emphasises the urgent need to increase opportunities for education and training to boost the number of EHC professionals in the region. Furthermore, the lack of information concerning the performance of EHC facilities within the health system highlights another critical area that requires further development in the EMR.
c) *Assessment of the capacity of the health sector to respond to current and future challenges*
  Despite the limited availability of detailed information specifically on the active health workforce for EHC services, valuable insights can still be obtained from the existing data on primary care facilities, physicians, pharmacists, nurses, and midwives. Health professionals belonging to these categories can contribute to EHC through various roles or task sharing arrangements. For instance, they can assist in screening for hearing loss and ear diseases, facilitating early interventions for these conditions. According to the literature, the workload of EHC professionals can be significantly reduced by 50 % if selected health interventions are undertaken by other healthcare professionals (Kamenov et al., 2021). This underscores the potential to optimise the capacity and efficiency of EHC services by involving a broader range of healthcare professionals in delivering certain aspects of EHC. Integrating packages of dental, vision, and hearing services, which have long been supported by the scientific community, can also be considered (Willink et al., 2017).
  By systematically training and building the capacity of general practitioners, nurses, and other healthcare workers, it is possible to facilitate hearing screening and early intervention (World Health Organization, 2021a). Additionally, this approach may contribute to effective surveillance of hearing loss outcomes at the population level.
d) *Assessment of service delivery models in the region*

Service delivery in the field of EHC can be significantly enhanced through the utilisation of telehealth and task-sharing approaches, applying evidence-based approaches for hearing aid provision in resource-limited settings (Dillard et al., 2024). Alternative service delivery models, such as telemedicine/tele-audiology, could work as sustainable solutions that can address ear and hearing health needs while improving geographical access (Bright et al., 2019, Bhutta, 2019, De Sousa et al., 2022, Swanepoel, 2020). These models could enable audiologists and trained non-specialists facilitators to administer hearing services, expanding the reach of care (Dillard et al., 2024).

To effectively implement hearing screening and integrate it into existing primary health services, more countries need to allocate resources and adequately finance EHC programs (World Health Organization, 2021b).The literature provides evidence on the estimated financial return on investment and the significant economic consequences of neglecting hearing loss, which strengthens the case for investing in EHC in the EMR (World Health Organization, 2021a, McDaid et al., 2021, McMahon et al., 2021).

### Implications for researchers, clinicians and policymakers in the EMR

The situational analysis presented in this study holds significant implications for researchers, clinicians, and policymakers. It is crucial to acknowledge the substantial societal and economic impact of unaddressed hearing loss (The Lancet Global Health, 2022, McDaid et al., 2021). The cost of unaddressed hearing loss in the EMR is substantial, amounting to nearly $30 billion per year, encompassing healthcare sector costs, educational sector costs, productivity losses, and societal costs related to social isolation and communication difficulties (eTable 7 in the Supplement).

To effectively respond to the current and future challenges, EHC should be integrated into national health policies and plans of individual countries. Investing in EHC has the potential to generate multiple benefits, with an estimated impact on addressing EHC problems for up to 65 million people by 2030. The scaling up of effective EHC services in the EMR is projected to result in significant benefits and productivity gains, with a net dollar return on investment estimated to be up to US$7 for every dollar invested (see eTable 8 in the Supplement). These findings highlight the potential for substantial positive outcomes and economic gains through the expansion and improvement of EHC services in the EMR region.

Compared to other WHO regions, the EMR faces unique challenges. The African Region, while projected to have the highest percentage increase in hearing loss (154.9 %), and other WHO regions have implemented various approaches to EHC service delivery. The World Report on Hearing highlights examples of successful hearing health initiatives across regions, including community-based models, task-sharing approaches, and digital health solutions. The EMR can learn from these global experiences while adapting interventions to its specific cultural, economic, and political contexts, including high rates of consanguineous marriages, ongoing conflicts, and diverse economic conditions (World Health Organization, 2021a).Based on the insights generated from the situational analysis in the EMR and aligned with the recommendations outlined in Chapter 4.7 of the *World report on hearing (*World Health Organization, 2021a*)*, the following actions should be undertaken by Ministries of Health in the EMR countries and territories to promote and enhance EHC in the region.

## Summary of suggested actions following the situation analysis

### Include EHC in universal health coverage

Actions:

a. Conduct a comprehensive situation assessment at the country/territory level to evaluate the current resources and readiness for EHC services. This assessment should encompass available facilities and can be facilitated through the use of the situation analysis tool developed by the World Health Organization (World Health Organization, 2015).
b. Incorporate people centred EHC into essential service packages within national health plans. This step ensures that EHC services are integrated and accessible to all individuals under the principles of universal health coverage, promoting equitable access.
c. Explore task redistribution strategies among existing health professionals, such as physicians, pharmacists, nurses, midwives, or trained community health workers. By sharing responsibilities, it is possible to enhance the capacity of the workforce and improve access to EHC services. This approach allows for more efficient use of human resources.
d. Develop the capacity to address current and future challenges effectively by leveraging digital health and technological solutions. These solutions can facilitate the delivery of interventions, enhance accessibility to healthcare, and support successful implementation in cases of task sharing. Embracing available digital health tools can greatly contribute to more accessible and efficient EHC services.

By implementing these actions, Ministries of Health in the EMR can make significant strides in promoting and enhancing EHC services throughout the region.

### Strengthen health systems to deliver EHC at all levels of care

Actions:

a. Establish EHC services at all levels of healthcare provision in an integrated manner, ensuring that the needs of all population groups are addressed. This approach guarantees equitable access to EHC services and ensures that individuals from diverse backgrounds can benefit from them.
b. Provide EHC services across the life course by integrating them into various programs and services. This includes incorporating screening for ear and hearing problems as part of child development programs, school health initiatives, occupational health services, programs for the care of older individuals, and health promotion activities. By integrating EHC into these programs, comprehensive care can be provided throughout individuals' lives.
c. Increase opportunities for education and training courses for EHC professionals and other healthcare professionals in the EMR. By expanding educational opportunities, the region can foster a public health approach among professionals, promoting a holistic understanding of EHC and its importance. This will contribute to building a skilled workforce and facilitate the implementation of effective EHC services.

Implementing these actions will ensure that EHC services are accessible and integrated across all levels of healthcare provision, spanning the entire life course. Furthermore, enhancing educational opportunities will contribute to building a competent workforce capable of delivering comprehensive EHC services in the EMR.

### Undertake awareness campaigns that address attitudes towards, and stigma related to, ear diseases and hearing loss

Raise awareness and inform the public about the preventable causes of hearing loss and ear diseases. This can be achieved through media campaigns and public health interventions that target individuals across the life-course. By disseminating accurate information, educating the public about the avoidable risk factors, and promoting healthy practices, the prevalence of hearing loss and ear diseases can be reduced.

Implementing these actions will ensure that EHC services are accessible and integrated across all levels of healthcare provision, spanning the entire life-course. Furthermore, enhancing educational opportunities will contribute to building a competent workforce capable of delivering comprehensive EHC services in the EMR. By raising awareness and providing information to the public, individuals can make informed decisions regarding their ear and hearing health, leading to the prevention of avoidable causes of hearing loss and ear diseases.

### Determine targets, monitor national trends, and evaluate progress

Actions:

a. Identify and incorporate comprehensive EHC indicators into the national health information systems. This involves integrating specific indicators that capture relevant data related to ear and hearing health. Relevant guidance on the analysis and use of routine health information systems was published by WHO in 2023 (World Health Organization, 2023b).
b. Monitor EHC indicators within public health surveillance systems, following the standard practices used for monitoring other non-communicable diseases' health indicators (Tsimpida et al., 2024). This ensures that EHC is given the same level of importance and attention as other health public health issues.

By incorporating comprehensive EHC indicators into national health information systems and surveillance systems, policymakers and healthcare providers will have access to reliable data for evidence-based decision-making, resource allocation, and the continuous improvement of EHC services.

### Promote high-quality public health research on EHC

Actions:

a. Enhance capacity to analyse and interpret epidemiological hearing data collected within the health information systems. By building research capacity, countries can generate valuable insights from the collected data, identify patterns, and understand the prevalence and impact of hearing loss and ear diseases within their populations.
b. Conduct research to investigate the association between hearing loss and other comorbid diseases, such as cardiovascular disease, stroke, diabetes, and others. By examining these links, countries can gain a better understanding of the current and future health challenges associated with hearing loss. This research can provide insights into shared risk factors, potential interventions, and strategies to improve public health outcomes. Understanding the relationship between hearing loss and comorbid diseases is essential for comprehensive healthcare planning and delivery.

It is important to acknowledge the significant resource and capacity constraints that many EMR countries face in implementing these recommendations. Political instability, competing health priorities, limited budgets, and workforce shortages present substantial challenges (World Health Organization,). A phased implementation approach prioritising high-impact, low-cost interventions may be most feasible in many EMR settings (World Health Organization, 2021a). Regional collaboration through resource sharing (such as joint procurement of hearing technologies, shared tele-audiology platforms, and pooled specialist services), joint training programs (including task-sharing protocols and sign language education), and structured knowledge exchange (documenting implementation successes and failures) could help individual countries overcome capacity and resource barriers. WHO and international NGOs could facilitate such cooperation through technical support, capacity building, and catalytic funding (World Health Organization, 2022).

However, realising these benefits requires understanding why widely known, evidence-based health system activities have not already been implemented across the region. This necessitates country-specific political economy analyses examining: what institutional, political, and economic factors shape health policy prioritisation; what constraints and barriers health planners face in practice; how competing health priorities are negotiated; and what implementation strategies have succeeded or failed in comparable settings. Only through such context-sensitive investigation can the region move from situation analysis to sustained improvements in EHC delivery.

### Strengths and limitations

This systematic review represents to our knowledge, the first comprehensive synthesis of evidence on the status of EHC in countries within the EMR. The study was structured according to the predefined themes of the WHO framework for national health policies, strategies, and plans (World Health Organization, 2010), providing a template for future updates and assessments.

An important methodological consideration is that our use of the WHO Building Blocks framework, while providing systematic structure for regional comparison, necessarily constrains our analysis to describing 'what' gaps exist rather than explaining 'why' they exist or 'how' to address them. The framework excels at providing a structured approach for identifying gaps and needs across the six essential health system components (leadership/governance, financing, workforce, medical products and technologies, information systems, and service delivery). This structured approach facilitates identification of common challenges requiring policy attention. However, the framework's normative structure also confines our analysis to predetermined categories of health system functioning. It does not, and is not designed to, analyse the underlying political economy factors, power dynamics, or institutional processes that explain why particular gaps exist or persist in different contexts. Neither does the framework prescribe specific pathways or implementation strategies for addressing identified shortcomings, as these are necessarily context-dependent and require country-specific analysis. Our study, therefore, serves as a situation analysis that maps the current EHC landscape and identifies where attention is needed, rather than as a policy analysis that explains causal mechanisms or recommends context-specific implementation approaches. For example, while we can document workforce shortages and financing gaps, the framework does not explain why some countries have prioritised EHC investment while others have not, or what political and institutional barriers impede policy implementation.

Another limitation is that some dimensions of EHC were more extensively covered in the literature than others, with a particular focus on current practices in hearing screening for newborns. Additionally, the majority of studies included in the analysis were published within the last decade, and the evidence primarily comes from 17 out of the 21 Member States of the EMR and the occupied Palestinian territory (including East Jerusalem). The limited representation and geographic focus of the studies introduce potential information bias and reduce the overall certainty of the evidence within the EMR (Harder et al., 2015).

Another significant limitation is language bias, as only English language studies were included in the review. This exclusion may have resulted in the omission of relevant evidence published in the native languages of the countries within the EMR. Furthermore, it is important to note that there may be alternative models of EHC service delivery established in certain countries, but these models were not captured in the review methodology due to the lack of published records.

Despite these limitations, this systematic review provides valuable insights into the current state of EHC in the EMR, highlighting areas where further research and data collection are needed. Our analysis may be subject to geographical bias, with some countries heavily represented (Iran with 46 studies, Saudi Arabia with 26 studies) while others are under-represented or absent entirely (no studies from Bahrain, Djibouti, Libya, Somalia). This uneven representation may skew our understanding of regional EHC challenges and limit the generalisability of findings across all EMR countries. Countries with ongoing conflicts or limited research infrastructure are particularly under-represented, potentially underestimating the true scope of EHC challenges in these settings. Additionally, while we identified alternative service delivery models such as tele-audiology and task-sharing as potential solutions, we did not conduct a detailed feasibility analysis of these approaches. Future research should critically evaluate the implementation feasibility, cost-effectiveness, and scalability of these innovative models across diverse EMR contexts, including infrastructure requirements, regulatory barriers, and cultural acceptability. Future efforts should also aim to address these limitations by including a wider range of countries, languages, and sources of evidence, thus improving the comprehensiveness and accuracy of future assessments of EHC in the region.

## Conclusion

This systematic review identified critical gaps in EHC across the Eastern Mediterranean Region (EMR), including insufficient epidemiological data, shortages of specialized professionals, limited screening programs beyond newborns, and underdeveloped rehabilitation services. While some countries have made notable progress in areas such as neonatal screening and early childhood screening (Djibouti, Iran), expanded EHC programs in primary healthcare units (Egypt), and mandatory neonatal hearing screening (Saudi Arabia), region-wide challenges persist in service accessibility, workforce capacity, and monitoring systems. The analysis revealed that consanguinity, genetic factors, otitis media, and noise exposure are the main risk factors for hearing loss in the region, with potentially 78.1 million people currently affected and projections suggesting this number could rise dramatically to 194 million by 2050 if not adequately addressed.

The findings of this situation analysis have led to the formulation of five key recommendations that can guide Member States of the EMR in developing a comprehensive strategy to address the current and future health challenges related to EHC. These include integrating EHC into universal health coverage, strengthening health systems to deliver EHC at all levels of care, undertaking awareness campaigns, establishing monitoring systems, and promoting high-quality research.

One of the most urgent needs identified is for robust and effective monitoring systems for EHC in the region. Incorporating comprehensive EHC indicators into national health information systems is crucial for tracking the prevalence, trends, and impact of ear and hearing health conditions, as well as evaluating the implementation and effectiveness of interventions.

The complex epidemiological profile of the EMR population, coupled with the significant burden of unaddressed hearing loss (estimated at $30 billion annually), highlights the importance of implementing national EHC policies, strategies, and plans. Alternative service delivery models such as telemedicine, task-sharing approaches, and integration of EHC into primary healthcare emerge as promising strategies to address the identified gaps, with potential return on investment estimated at up to US$7 for every dollar invested.

By addressing the gaps identified in this situation analysis and implementing the recommended strategies, Member States of the EMR can make significant progress in enhancing EHC services and improving the ear and hearing health outcomes of their populations. The findings of this paper serve as a valuable resource for policymakers, healthcare professionals, and stakeholders in shaping the future of EHC in the EMR.It is important to acknowledge the significant resource and capacity constraints that many EMR countries face in implementing these recommendations. Political instability, competing health priorities, limited budgets, and workforce shortages present substantial challenges. The heterogeneous nature of the region means that a one-size-fits-all approach is inadequate. High-income countries in the region may focus on advanced technological solutions and specialised workforce development, while low-income and conflict-affected areas require fundamental infrastructure development and basic service delivery models.

A phased implementation approach prioritising high-impact, low-cost interventions may be most feasible. Regional collaboration through resource sharing, joint training programs, and knowledge exchange could help overcome some of these barriers. WHO and international NGOs could facilitate such cooperation through technical support, capacity building, and catalytic funding. Understanding why these widely known health system activities have not been implemented requires examining country-specific political, economic, and social contexts that influence health policy prioritisation and resource allocation.

This situational analysis serves two critical functions. First, it demonstrates the urgent need for national policymakers and health planners in EMR countries and territories to incorporate EHC systematically into their health policy frameworks, resource allocation decisions, and service delivery plans. The identification of substantial gaps - in epidemiological surveillance, workforce capacity, financing mechanisms, and service coverage - provides a clear mandate for elevating EHC on national health agendas. Without deliberate policy attention and adequate resource allocation, the projected increase in hearing loss burden (from 22.1 million with disabling hearing loss in 2020–51.7 million by 2050) will overwhelm already strained health systems.

Second, this review establishes the foundation for a necessary programme of country-specific implementation research. While our regional analysis identifies common challenges and provides some comparative data, the heterogeneity across EMR countries demands localised investigation. Future research must examine: why evidence-based EHC interventions have not been implemented despite their known effectiveness; what specific political, economic, institutional, and cultural barriers exist in different country contexts; how EHC can be integrated within existing health system structures and competing priorities; and what implementation strategies are most effective and sustainable across diverse settings ranging from high-income countries to conflict-affected territories. Country-by-country studies involving local researchers, clinicians, policymakers, and affected communities are essential to translate this regional situation analysis into actionable, context-specific health system reforms. Such investigations will build upon our regional situation analysis to develop contextualised strategies that account for local governance structures, financing mechanisms, cultural factors, conflict situations, and existing health system capacities.

## CRediT authorship contribution statement

**Dialechti Tsimpida:** Writing – review & editing, Writing – original draft, Visualization, Validation, Software, Resources, Project administration, Methodology, Investigation, Formal analysis, Data curation, Conceptualization. **Hala Sakr:** Writing – review & editing, Supervision, Methodology, Investigation, Conceptualization. **Abdelrahman Elwishahy:** Writing – review & editing, Methodology, Investigation, Conceptualization. **Shelly Chadha:** Writing – review & editing, Supervision, Methodology, Investigation, Conceptualization. **Chander Chitra:** Writing – review & editing, Methodology, Investigation, Conceptualization. **Saied Mahmoudian:** Writing – review & editing, Methodology, Investigation, Formal analysis, Conceptualization.

## Funding

This research did not receive any specific grant from funding agencies in the public, commercial, or not-for-profit sectors.

## Declaration of Competing Interest

The authors declare that they have no known competing financial interests or personal relationships that could have appeared to influence the work reported in this article.
