# Supplementary material for: Situational analysis of health systems for ear and hearing care in the World Health Organization (WHO) Eastern Mediterranean Region: A systematic review and evidence synthesis to inform national policies and strategies
Source: SSM Health Syst. 2026 Jun;6:100170. doi: 10.1016/j.ssmhs.2026.100170 (PMC13176227; doi:10.1016/j.ssmhs.2026.100170)
Supplement: Supplementary file 1 — Supplementary material [file mmc1.docx]

**Supporting information captions**

**S1 Table.** Completed PRISMA checklist

**S2 Table.** Summary of the quality appraisal of included studies

**S3 Table.** Population size (in thousands) in countries and territories of the Eastern Mediterranean Region (EMR)

**S4 Table.** Net primary school enrolment ratio per 100 school-age children in countries and territories of the Eastern Mediterranean Region (EMR)

**S5 Table.** Burden of disease (measured as deaths per 100,000) in countries and territories of the WHO Eastern Mediterranean Region in 2019, for both sexes, and all ages

**S6 Table.** Country-specific information on newborn infant hearing screening, as retrieved by informant replies

**S7 Table.** Combined direct, indirect and intangible costs of hearing loss in WHO Eastern Mediterranean Region (in billion dollars)

**S8 Table.** Expected impact of investing in Ear and Hearing Care (EHC) in WHO Eastern Mediterranean Region (Cost in US$)

**S1 Methods.** Database-specific search strategies

**S1 Table.** PRISMA 2020 item checklist

| **Section and Topic** | **Item #** | **Checklist item** | **Location where item is reported** |
| --- | --- | --- | --- |
| **TITLE** | | |  |
| Title | 1 | Identify the report as a systematic review. | p.1 |
| **ABSTRACT** | | |  |
| Abstract | 2 | See the PRISMA 2020 for Abstracts checklist. | p.2-3 |
| **INTRODUCTION** | | |  |
| Rationale | 3 | Describe the rationale for the review in the context of existing knowledge. | p.2-4 |
| Objectives | 4 | Provide an explicit statement of the objective(s) or question(s) the review addresses. | p.4 |
| **METHODS** | | |  |
| Eligibility criteria | 5 | Specify the inclusion and exclusion criteria for the review and how studies were grouped for the syntheses. | p.5-6 |
| Information sources | 6 | Specify all databases, registers, websites, organisations, reference lists and other sources searched or consulted to identify studies. Specify the date when each source was last searched or consulted. | p.5 |
| Search strategy | 7 | Present the full search strategies for all databases, registers and websites, including any filters and limits used. | eMethods 1 in the Supplement |
| Selection process | 8 | Specify the methods used to decide whether a study met the inclusion criteria of the review, including how many reviewers screened each record and each report retrieved, whether they worked independently, and if applicable, details of automation tools used in the process. | p.7 |
| Data collection process | 9 | Specify the methods used to collect data from reports, including how many reviewers collected data from each report, whether they worked independently, any processes for obtaining or confirming data from study investigators, and if applicable, details of automation tools used in the process. | p.6-7 |
| Data items | 10a | List and define all outcomes for which data were sought. Specify whether all results that were compatible with each outcome domain in each study were sought (e.g. for all measures, time points, analyses), and if not, the methods used to decide which results to collect. | p.7-8 |
|  | 10b | List and define all other variables for which data were sought (e.g. participant and intervention characteristics, funding sources). Describe any assumptions made about any missing or unclear information. | p.7-8 |
| Study risk of bias assessment | 11 | Specify the methods used to assess risk of bias in the included studies, including details of the tool(s) used, how many reviewers assessed each study and whether they worked independently, and if applicable, details of automation tools used in the process. | p.8 |
| Effect measures | 12 | Specify for each outcome the effect measure(s) (e.g. risk ratio, mean difference) used in the synthesis or presentation of results. | n/a |
| Synthesis methods | 13a | Describe the processes used to decide which studies were eligible for each synthesis (e.g. tabulating the study intervention characteristics and comparing against the planned groups for each synthesis (item #5)). | p.8 |
|  | 13b | Describe any methods required to prepare the data for presentation or synthesis, such as handling of missing summary statistics, or data conversions. | p.8-9 |
|  | 13c | Describe any methods used to tabulate or visually display results of individual studies and syntheses. | p.8-9 |
|  | 13d | Describe any methods used to synthesize results and provide a rationale for the choice(s). If meta-analysis was performed, describe the model(s), method(s) to identify the presence and extent of statistical heterogeneity, and software package(s) used. | p.8-9 |
|  | 13e | Describe any methods used to explore possible causes of heterogeneity among study results (e.g. subgroup analysis, meta-regression). | n/a |
|  | 13f | Describe any sensitivity analyses conducted to assess robustness of the synthesized results. | n/a |
| Reporting bias assessment | 14 | Describe any methods used to assess risk of bias due to missing results in a synthesis (arising from reporting biases). | p.9 |
| Certainty assessment | 15 | Describe any methods used to assess certainty (or confidence) in the body of evidence for an outcome. | p.9 |
| **RESULTS** | | |  |
| Study selection | 16a | Describe the results of the search and selection process, from the number of records identified in the search to the number of studies included in the review, ideally using a flow diagram. | Figure1 |
|  | 16b | Cite studies that might appear to meet the inclusion criteria, but which were excluded, and explain why they were excluded. | Figure1 |
| Study characteristics | 17 | Cite each included study and present its characteristics. | Table 1 |
| Risk of bias in studies | 18 | Present assessments of risk of bias for each included study. | eTable 2 in the Supplement |
| Results of individual studies | 19 | For all outcomes, present, for each study: (a) summary statistics for each group (where appropriate) and (b) an effect estimate and its precision (e.g. confidence/credible interval), ideally using structured tables or plots. | Tables 1-4 |
| Results of syntheses | 20a | For each synthesis, briefly summarise the characteristics and risk of bias among contributing studies. | eTable 2 in the Supplement |
|  | 20b | Present results of all statistical syntheses conducted. If meta-analysis was done, present for each the summary estimate and its precision (e.g. confidence/credible interval) and measures of statistical heterogeneity. If comparing groups, describe the direction of the effect. | Tables 1-4 |
|  | 20c | Present results of all investigations of possible causes of heterogeneity among study results. | n/a |
|  | 20d | Present results of all sensitivity analyses conducted to assess the robustness of the synthesized results. | n/a |
| Reporting biases | 21 | Present assessments of risk of bias due to missing results (arising from reporting biases) for each synthesis assessed. | p.29 |
| Certainty of evidence | 22 | Present assessments of certainty (or confidence) in the body of evidence for each outcome assessed. | eTable 2 in the Supplement |
| **DISCUSSION** | | |  |
| Discussion | 23a | Provide a general interpretation of the results in the context of other evidence. | p.21-28 |
|  | 23b | Discuss any limitations of the evidence included in the review. | p.26-27 |
|  | 23c | Discuss any limitations of the review processes used. | p.27-28 |
|  | 23d | Discuss implications of the results for practice, policy, and future research. | p. 21-30 |
| **OTHER INFORMATION** | | |  |
| Registration and protocol | 24a | Provide registration information for the review, including register name and registration number, or state that the review was not registered. | p.5 |
|  | 24b | Indicate where the review protocol can be accessed, or state that a protocol was not prepared. | p.5 |
|  | 24c | Describe and explain any amendments to information provided at registration or in the protocol. | n/a |
| Support | 25 | Describe sources of financial or non-financial support for the review, and the role of the funders or sponsors in the review. | Described |
| Competing interests | 26 | Declare any competing interests of review authors. | Declared |
| Availability of data, code and other materials | 27 | Report which of the following are publicly available and where they can be found: template data collection forms; data extracted from included studies; data used for all analyses; analytic code; any other materials used in the review. | Search strategy included in the Supplement |

*From:*  Page MJ, McKenzie JE, Bossuyt PM, Boutron I, Hoffmann TC, Mulrow CD, et al. The PRISMA 2020 statement: an updated guideline for reporting systematic reviews. BMJ 2021;372:n71. doi: 10.1136/bmj.n71

For more information, visit: <http://www.prisma-statement.org/>

**S2 Table.** Summary of the quality appraisal of included studies

| **No** | **Country** | **Bibliography** | Selection bias^a^ | Epidemiological study design^b^ | Covariates^c^ | Data collection methods^d^ | Total quality score^e^ |
| --- | --- | --- | --- | --- | --- | --- | --- |
| 1 | Afghanistan | (Nasir et al., 2004) | 0 | 1 | 0 | 1 | 2 |
|  | Bahrain | **-** |  |  |  |  |  |
|  | Djibouti | **-** |  |  |  |  |  |
| 2 | Egypt | (Elshaer et al., 2023) | 1 | 1 | 1 | 1 | 4 |
| 3 | Egypt | (M. M. Taha et al., 2023) | 0 | 0 | 0 | 1 | 1 |
| 4 | Egypt | (Gibriel et al., 2019) | 0 | 1 | 0 | 1 | 2 |
| 5 | Egypt | (Khairy et al., 2018) | 0 | 1 | 0 | 1 | 2 |
| 6 | Egypt | (El-Badry et al., 2014) | 0 | 1 | 0 | 1 | 2 |
| 7 | Egypt | (Yamamah et al., 2012) | 0 | 0 | 0 | 1 | 1 |
| 8 | Egypt | (A. A. Taha et al., 2010) | 0 | 1 | 1 | 1 | 3 |
| 9 | Egypt | (Sanyelbhaa Talaat et al., 2009) | 0 | 1 | 0 | 1 | 2 |
| 10 | Egypt | (Abdel Hamid et al., 2007) | 1 | 1 | 1 | 1 | 4 |
| 11 | Iran (Islamic Republic of) | (Mohseni et al., 2025) | 0 | 1 | 0 | 1 | 2 |
| 12 | Iran (Islamic Republic of) | (Esmaeili et al., 2025) | 1 | 0 | 1 | 1 | 3 |
| 13 | Iran (Islamic Republic of) | Nadri et al., 2024) | 0 | 0 | 0 | 1 | 1 |
| 14 | Iran (Islamic Republic of) | (Ghosn et al., 2024) | 0 | 0 | 1 | 1 | 2 |
| 15 | Iran (Islamic Republic of) | (M. Asghari et al., 2024) | 0 | 0 | 1 | 1 | 2 |
| 16 | Iran (Islamic Republic of) | (Sudani et al., 2024) | 1 | 0 | 1 | 1 | 3 |
| 17 | Iran (Islamic Republic of) | (Aliazami et al., 2023) | n/a | n/a | n/a | n/a | Review |
| 18 | Iran (Islamic Republic of) | (Etemadinezhad et al., 2023) | n/a | n/a | n/a | n/a | Review |
| 19 | Iran (Islamic Republic of) | (Jafarzadeh et al., 2023) | 1 | 1 | 1 | 1 | 4 |
| 20 | Iran (Islamic Republic of) | (Mohammadi et al., 2023) | 0 | 0 | 1 | 1 | 2 |
| 21 | Iran (Islamic Republic of) | (Rahimi et al., 2023) | 1 | 0 | 1 | 1 | 3 |
| 22 | Iran (Islamic Republic of) | (Vallian Broojeni et al., 2023) | 1 | 0 | 0 | 1 | 2 |
| 23 | Iran (Islamic Republic of) | (Afshar et al., 2022) | 0 | 1 | 0 | 1 | 2 |
| 24 | Iran (Islamic Republic of) | (Babanejad et al., 2022) | n/a | n/a | n/a | n/a | Review |
| 25 | Iran (Islamic Republic of) | (Gharibi & Khavidaki, 2022) | 1 | 0 | 1 | 1 | 3 |
| 26 | Iran (Islamic Republic of) | (Golbabaei Pasandi et al., 2022) | 1 | 1 | 1 | 1 | 4 |
| 27 | Iran (Islamic Republic of) | (Saraei et al., 2022) | 1 | 0 | 1 | 1 | 3 |
| 28 | Iran (Islamic Republic of) | (Mahmoudian et al., 2021) | n/a | n/a | n/a | n/a | Review |
| 29 | Iran (Islamic Republic of) | (Moradi et al., 2021) | n/a | n/a | n/a | n/a | Review |
| 30 | Iran (Islamic Republic of) | (Jalali et al., 2020) | 0 | 1 | 1 | 1 | 3 |
| 31 | Iran (Islamic Republic of) | (Koohiyan, 2020) | n/a | n/a | n/a | n/a | Review |
| 32 | Iran (Islamic Republic of) | (Hajilari et al., 2019) | 1 | 1 | 0 | 1 | 3 |
| 33 | Iran (Islamic Republic of) | (koohiyan, 2019) | n/a | n/a | n/a | n/a | Review |
| 34 | Iran (Islamic Republic of) | (Monshizadeh et al., 2019) | 0 | 1 | 0 | 1 | 2 |
| 35 | Iran (Islamic Republic of) | (Keihanidost et al., 2018) | 0 | 1 | 0 | 1 | 2 |
| 36 | Iran (Islamic Republic of) | (Saffari et al., 2018) | 0 | 1 | 0 | 1 | 2 |
| 37 | Iran (Islamic Republic of) | (Zahed Pasha et al., 2018) | 0 | 1 | 0 | 1 | 2 |
| 38 | Iran (Islamic Republic of) | (A. Asghari et al., 2017) | 1 | 0 | 1 | 1 | 3 |
| 39 | Iran (Islamic Republic of) | (Ghasemnejad et al., 2017) | n/a | n/a | n/a | n/a | Review |
| 40 | Iran (Islamic Republic of) | (Saki et al., 2017) | 1 | 0 | 0 | 1 | 2 |
| 41 | Iran (Islamic Republic of) | (Beheshtian et al., 2016) | n/a | n/a | n/a | n/a | Review |
| 42 | Iran (Islamic Republic of) | (Tajik & Ahmadpour-kacho, 2016) | 0 | 1 | 0 | 1 | 2 |
| 43 | Iran (Islamic Republic of) | (Alaee et al., 2015) | 0 | 0 | 0 | 1 | 1 |
| 44 | Iran (Islamic Republic of) | (Daneshi et al., 2015) | 0 | 1 | 0 | 1 | 2 |
| 45 | Iran (Islamic Republic of) | (Farhat et al., 2015) | 0 | 1 | 0 | 1 | 2 |
| 46 | Iran (Islamic Republic of) | (Firoozbakht et al., 2014) | 1 | 1 | 0 | 1 | 3 |
| 47 | Iran (Islamic Republic of) | (Haghshenas et al., 2014) | 0 | 1 | 0 | 1 | 2 |
| 48 | Iran (Islamic Republic of) | (Jeddi et al., 2014) | 0 | 0 | 0 | 1 | 1 |
| 49 | Iran (Islamic Republic of) | (Panahi et al., 2014) | 0 | 0 | 0 | 1 | 1 |
| 50 | Iran (Islamic Republic of) | (Baradaranfar et al., 2011) | 0 | 1 | 0 | 1 | 2 |
| 51 | Iran (Islamic Republic of) | (Mohammadzadeh et al., 2011) | 0 | 1 | 0 | 1 | 2 |
| 52 | Iran (Islamic Republic of) | (Mahdieh et al., 2010) | n/a | n/a | n/a | n/a | Review |
| 53 | Iran (Islamic Republic of) | (Sarafraz & Ahmadi, 2009) | 0 | 0 | 0 | 1 | 1 |
| 54 | Iran (Islamic Republic of) | (Jafari et al., 2007) | 0 | 0 | 0 | 1 | 1 |
| 55 | Iran (Islamic Republic of) | (Lotfi & Mehrkian, 2007) | 0 | 0 | 0 | 1 | 1 |
| 56 | Iran (Islamic Republic of) | (Pouryaghoub et al., 2007) | 0 | 0 | 0 | 1 | 1 |
| 57 | Iraq | (Al Samarrai et al., 2024) | 1 | 0 | 0 | 1 | 2 |
| 58 | Iraq | (Al-Obeidy et al., 2019) | 0 | 1 | 0 | 1 | 2 |
| 59 | Jordan | (Obeidat et al, 2024) | 1 | 1 | 1 | 1 | 4 |
| 60 | Jordan | (Almaayeh et al., 2018) | 0 | 1 | 0 | 1 | 2 |
| 61 | Jordan | (Al-Dababneh et al., 2016) | 0 | 0 | 1 | 0 | 1 |
| 62 | Jordan | (Abu-Shaheen et al., 2014) | 1 | 0 | 1 | 1 | 3 |
| 63 | Jordan | (Attias et al., 2006) | 1 | 1 | 0 | 1 | 3 |
| 64 | Jordan | (Medlej-Hashim et al., 2002) | 0 | 1 | 0 | 1 | 2 |
| 65 | Kuwait | (Al-Kandari & Alshuaib, 2010) | 0 | 1 | 0 | 1 | 2 |
| 66 | Kuwait | (Al-Kandari & Alshuaib, 2007) | 0 | 1 | 0 | 1 | 2 |
| 67 | Lebanon | (Fooladi, 2012) | 0 | 0 | 0 | 1 | 1 |
| 68 | Lebanon | (El Zir et al., 2008) | 0 | 0 | 0 | 1 | 1 |
| 69 | Lebanon | (Tabchi et al., 2000) | 0 | 1 | 0 | 1 | 2 |
|  | Libya | **-** |  |  |  |  |  |
| 70 | Morocco | (El Fizazi et al., 2024) | 1 | 1 | 0 | 1 | 3 |
| 71 | Morocco | (AitRaise et al., 2023) | 0 | 0 | 0 | 1 | 1 |
| 72 | Oman | (Khandekar et al., 2010) | 0 | 0 | 1 | 1 | 2 |
| 73 | Oman | (Khabori & Patton, 2008) | 1 | 1 | 0 | 1 | 3 |
| 74 | Oman | (Khabori & Khandekar, 2007) | 1 | 0 | 0 | 1 | 2 |
| 75 | Oman | (Khandekar et al., 2006) | 1 | 1 | 0 | 1 | 3 |
| 76 | Oman | (Al Khabori & Khandekar, 2004) | 1 | 0 | 0 | 1 | 2 |
| 77 | Oman | (Al Khabori, 2004) | 1 | 1 | 0 | 1 | 3 |
| 78 | Pakistan | (Shadab et al., 2024) | n/a | n/a | n/a | n/a | Review |
| 79 | Pakistan | (Naz, 2022) | n/a | n/a | n/a | n/a | Review |
| 80 | Pakistan | (J. Ahmed et al., 2020) | 0 | 1 | 0 | 1 | 2 |
| 81 | Pakistan | (Doll et al., 2020) | 0 | 0 | 0 | 1 | 1 |
| 82 | Pakistan | (Zhou et al., 2020) | 0 | 0 | 0 | 1 | 1 |
| 83 | Pakistan | (Mumtaz & Saqulain, 2020) | 0 | 0 | 0 | 0 | 0 |
| 84 | Pakistan | (Mumtaz et al., 2019) | 0 | 0 | 0 | 0 | 0 |
| 85 | Pakistan | (Richard et al., 2019) | 0 | 0 | 0 | 1 | 1 |
| 86 | Pakistan | (S. Ahmed et al., 2018) | 0 | 1 | 0 | 1 | 2 |
| 87 | Pakistan | (Wasim et al., 2018) | n/a | n/a | n/a | n/a | Review |
| 88 | Pakistan | (Qureshi et al., 2017) | 0 | 1 | 0 | 1 | 2 |
| 89 | Pakistan | (Mustafa et al., 2017) | 1 | 1 | 0 | 1 | 3 |
| 90 | Pakistan | (Naz et al., 2017) | 0 | 1 | 0 | 1 | 2 |
| 91 | Pakistan | (Shakoor et al., 2016) | 1 | 1 | 0 | 1 | 3 |
| 92 | Pakistan | (Halim & Abbas, 2015) | 0 | 0 | 0 | 1 | 1 |
| 93 | Pakistan | (Salman et al., 2015) | 0 | 0 | 0 | 1 | 1 |
| 94 | Pakistan | (Yan et al., 2015) | n/a | n/a | n/a | n/a | Review |
| 95 | Pakistan | (Ibrahim & Bhutta, 2013) | 1 | 1 | 1 | 1 | 4 |
| 96 | Pakistan | (Raza et al., 2012) | 0 | 1 | 1 | 1 | 3 |
| 97 | Pakistan | (Musani et al., 2011) | 0 | 1 | 0 | 1 | 2 |
| 98 | Pakistan | (Sajjad et al., 2008) | 0 | 1 | 1 | 1 | 3 |
| 99 | Pakistan | (Khan et al., 2007) | 0 | 0 | 0 | 1 | 1 |
| 100 | Pakistan | (O’Hara et al., 2002) | 0 | 1 | 0 | 1 | 2 |
| 101 | Palestine | (Shehabi et al., 2023) | 0 | 1 | 1 | 1 | 3 |
| 102 | Palestine | (Corradin et al., 2014) | 0 | 1 | 0 | 1 | 2 |
| 103 | Qatar | (Alkhidir et al., 2024) | 1 | 1 | 1 | 1 | 4 |
| 104 | Qatar | (Girotto et al., 2014) | n/a | n/a | n/a | n/a | Review |
| 105 | Qatar | (Bener et al., 2005) | 0 | 1 | 1 | 1 | 3 |
| 106 | Saudi Arabia | (Alnoury et al., 2025) | 0 | 0 | 0 | 1 | 1 |
| 107 | Saudi Arabia | (Aljabri et al., 2025) | n/a | n/a | n/a | n/a | Review |
| 108 | Saudi Arabia | Al-Shaikh, 2024) | 1 | 1 | 0 | 1 | 3 |
| 109 | Saudi Arabia | (Alanazi et al., 2024) | 1 | 0 | 0 | 1 | 2 |
| 110 | Saudi Arabia | (Almalki, 2024) | n/a | n/a | n/a | n/a | Review |
| 111 | Saudi Arabia | (Alothman et al., 2024) | 1 | 0 | 0 | 1 | 2 |
| 112 | Saudi Arabia | (Alateeq et al., 2023) | 1 | 0 | 1 | 1 | 3 |
| 113 | Saudi Arabia | (Alzahrani et al., 2023) | 1 | 0 | 0 | 0 | 1 |
| 114 | Saudi Arabia | (ALqarny et al., 2021) | 1 | 1 | 0 | 1 | 3 |
| 115 | Saudi Arabia | (Alasim, 2020) | 0 | 0 | 0 | 1 | 1 |
| 116 | Saudi Arabia | (H. N. Fageeh & Mansoor, 2020) | 0 | 1 | 0 | 1 | 2 |
| 117 | Saudi Arabia | (Alkahtani et al., 2019) | 0 | 0 | 1 | 1 | 2 |
| 118 | Saudi Arabia | (Halawani et al., 2019) | 0 | 1 | 0 | 1 | 2 |
| 119 | Saudi Arabia | (Alharbi & Ahmed, 2015) | 0 | 1 | 0 | 1 | 2 |
| 120 | Saudi Arabia | (Al-Mazrou et al., 2014) | 0 | 1 | 0 | 1 | 2 |
| 121 | Saudi Arabia | (Al-Rowaily et al., 2012) | 0 | 0 | 0 | 1 | 1 |
| 122 | Saudi Arabia | (Al-Muhaimeed et al., 2009) | 0 | 1 | 0 | 1 | 2 |
| 123 | Saudi Arabia | (Habib & Abdelgaffar, 2005) | 1 | 1 | 0 | 1 | 3 |
| 124 | Saudi Arabia | (H. O. Ahmed et al., 2004) | 0 | 0 | 0 | 1 | 1 |
| 125 | Saudi Arabia | (Maisoun & Zakzouk, 2003) | 0 | 0 | 0 | 1 | 1 |
| 126 | Saudi Arabia | (Al-Abduljawad & Zakzouk, 2003) | 1 | 0 | 0 | 1 | 2 |
| 127 | Saudi Arabia | (N. A. Fageeh, 2004) | 0 | 0 | 0 | 1 | 1 |
| 128 | Saudi Arabia | (Daghistani et al., 2002) | 0 | 0 | 0 | 1 | 1 |
| 129 | Saudi Arabia | (Zakzouk et al., 2002) | 1 | 1 | 1 | 1 | 4 |
| 130 | Saudi Arabia | (Zakzouk & Hajjaj, 2002) | 1 | 0 | 0 | 1 | 2 |
| 131 | Saudi Arabia | (H. O. Ahmed et al., 2001) | 1 | 1 | 0 | 1 | 3 |
|  | Somalia | **-** |  |  |  |  |  |
| 132 | Sudan | (Alier et al., 2025) | 0 | 0 | 0 | 1 | 1 |
| 133 | Sudan | (S. Ahmed et al., 2017) | 0 | 1 | 0 | 1 | 2 |
| 134 | Syrian Arab Republic | (Yücel et al., 2019) | 0 | 0 | 0 | 1 | 1 |
| 135 | Syrian Arab Republic | (Kaheel et al., 2018) | 0 | 1 | 0 | 1 | 2 |
| 136 | Syrian Arab Republic | (Moassass et al., 2018) | 0 | 0 | 0 | 1 | 1 |
| 137 | Tunisia | (Romdhane et al., 2014) | n/a | n/a | n/a | n/a | Review |
| 138 | Tunisia | (Abed et al., 2013) | 0 | 1 | 0 | 1 | 2 |
| 139 | Tunisia | (Nouaili et al., 2010) | 0 | 1 | 0 | 1 | 2 |
| 140 | Tunisia | (Ben Arab et al., 2004) | 1 | 1 | 0 | 1 | 3 |
| 141 | United Arab Emirates | (Elsayed & Al‐Shamsi, 2022) | 1 | 1 | 0 | 1 | 3 |
| 142 | United Arab Emirates | (Tlili et al., 2017) | 0 | 0 | 0 | 1 | 1 |
| 143 | United Arab Emirates | (Borders et al., 2016) | n/a | n/a | n/a | n/a | Review |
| 144 | United Arab Emirates | (Ur Rehman et al., 2012) | 0 | 1 | 0 | 1 | 2 |
| 145 | Yemen | (Asaad et al., 2023) | 0 | 1 | 0 | 1 | 2 |
| 146 | Yemen | (Al’shardzhabi & Tsygankova, 2014) | 0 | 1 | 0 | 1 | 2 |

Each point given indicates the presence of the relevant criterion

^a^ Selection bias: likely to be representative of the target population at the country level and have a response rate or data capture among eligible participants of 70% or greater.

^b^ Epidemiological study design: cohort study, case-control study, ecological study, or interrupted time series.

^c^ Covariates: control for a minimum of 2 critical covariates in the analysis, including socioeconomic characteristics (e.g., education, occupation, income, wealth).

^d^ Data collection methods: psychoacoustic hearing assessment tools, which are valid and reliable, or data from medical records.

^e^ Each point given for the quality of evidence indicates the presence of the relevant criterion, and articles were rated as high (4 points), moderate (3 points), low (2 point) and very low (1 point or 0 points).

**S3 Table.** Population size (in thousands) in countries and territories of the Eastern Mediterranean Region (EMR)


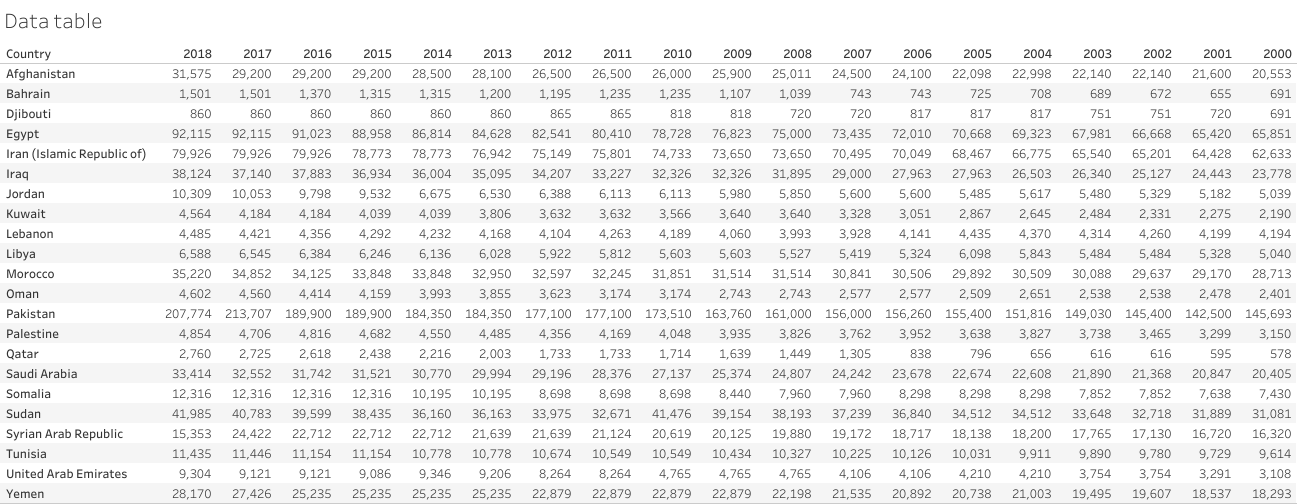


Data source: WHO regional office of the Eastern Mediterranean (last update: June 19, 2020)

**S4 Table.** Net primary school enrolment ratio per 100 school-age children in countries and territories of the Eastern Mediterranean Region (EMR)


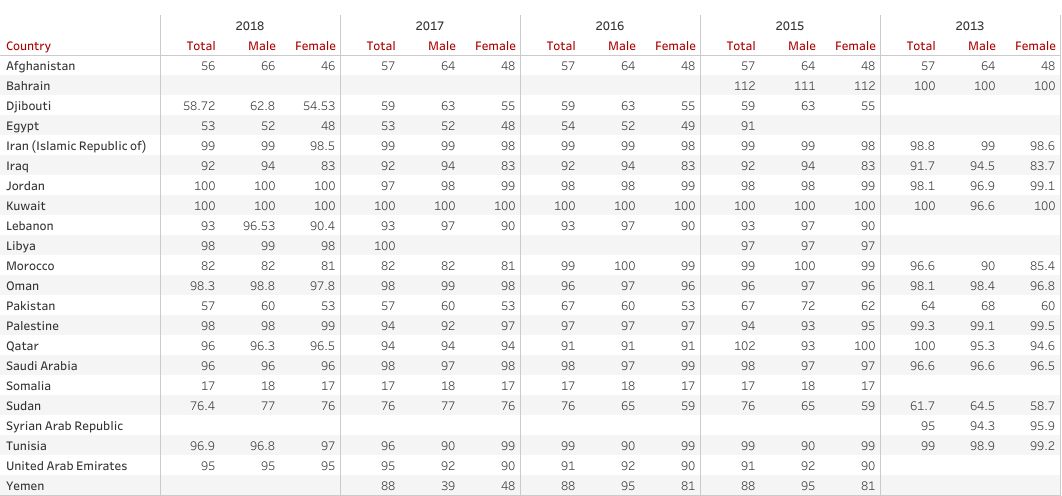


Data source: WHO regional office of the Eastern Mediterranean (last update: June 18, 2020)

**S5 Table.**  Burden of disease (measured as deaths per 100,000) in countries and territories of the WHO Eastern Mediterranean Region in 2019, for both sexes, and all ages


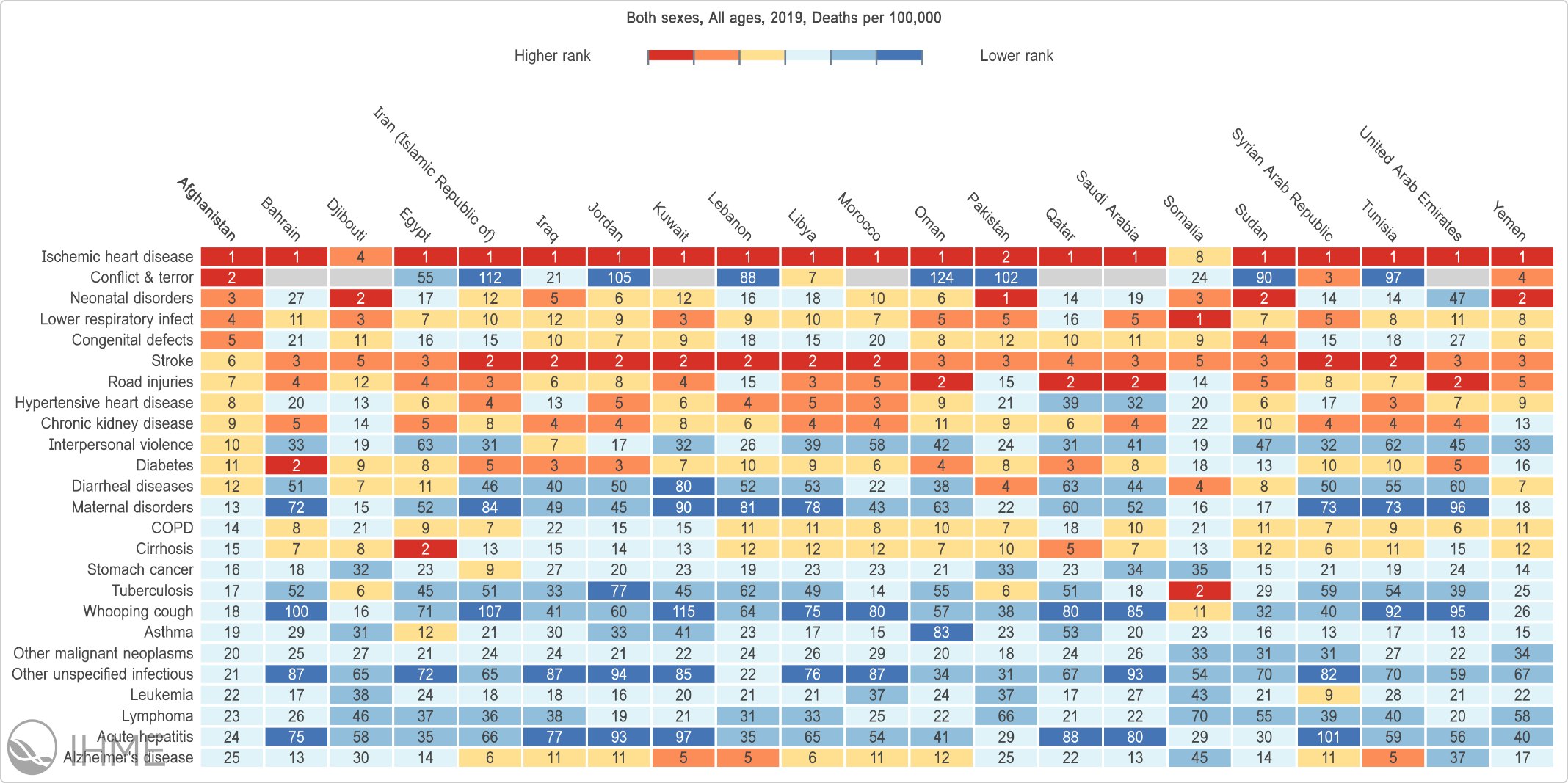


Data were synthesised using GBD Compare (<https://vizhub.healthdata.org/gbd-compare/>)

**S6 Table.** Country-specific information on newborn infant hearing screening, as retrieved by informant replies*

| **Country** | **Reporting year: data on life births from national statistical institutes and state welfare organisations** | **% of newborns screened: babies with PCHL**  **per 1000** | **Method used (%)** | **Screening mandated?** | **Where was screening done?** | **Who screened?** |
| --- | --- | --- | --- | --- | --- | --- |
| Afghanistan^a^ | - |  |  |  |  |  |
| Bahrain^a^ | - |  |  |  |  |  |
| Djibouti^b^ | - |  |  |  |  |  |
| Egypt^a^ | - |  |  |  |  |  |
| Iran (Islamic Republic of) | 2013: 1,427,653 | 66.4%, UNHS, 3.4% targeted: 2.6 | 20% OAE, 80% OAE-AABR | No | 95% birth facilities, 5% outpatient centers | 65% audiologists, 10% nurses, 5% midwives, 20% community health workers |
| Iraq | 2015 & 2018: about 1,079,000 | Sporadic: 0.5 | 100% OAE | Yes, UNHS/2017 | 100% audiology centers | 100% physicians |
| Jordan | 2013: 178,000 | 68%, 67% UNHS, 1% targeted: 6.0 | 99% OAE,  1% AABR | No | 90% birth facilities, 10% other | 30% audiologists, 70% nurses |
| Kuwait | 2014: 61,313 | UNHS started in 2013 in 5 hospitals |  | Not reported |  |  |
| Lebanon^a^ | - |  |  |  |  |  |
| Libya^b^ | - |  |  |  |  |  |
| Morocco^b^ | - |  |  |  |  |  |
| Oman | 2014: 71,650 | 96,6% UNHS, targeted 0.04%: 1.0 | 100% OAE | Yes, UNHS/1996 | 99% birth facilities, 1% mother–child clinics | nurses, 90% midwives |
| Pakistan | 2013: 4,666,000 | 2% UNHS, 3% later, 2% targeted: 15.0 | 70% OAE, 5% AABR, 25% OAEA ABR | No | 10% birth facilities, 90% audiology clinics | 100% audiologists |
| Palestine | 2011: 121,493 | no UNHS, 3% later | 97% OAE, 3% AABR | No | birth facilities, outpatient | 100% nurses |
| Qatar | 2012: 18,067 | 97% UNHS: 1.8 | 100% OAE-AABR | Yes, UNHS/2003 | 100% birth facilities, private clinics | 100% audio-physicians |
| Saudi Arabia | 2014: 569,000 | hospital-based UNHS or targeted: 1.8 |  | Yes | not reported | not reported |
| Somalia^b^ | - |  |  |  |  |  |
| Sudan^a^ | - |  |  |  |  |  |
| Syrian Arab Republic^b^ | - |  |  |  |  |  |
| Tunisia^a^ | - |  |  |  |  |  |
| United Arab Emirates^b^ | - |  |  |  |  |  |
| Yemen^a^ | - |  |  |  |  |  |

* Data based on the survey on the global status of newborn and infant hearing screening (Neumann et al., 2020)

^a^No information was obtained from the following countries ^b^Newborn infant hearing screening was not yet established in the reporting period

Acronyms: AABR = automated auditory brainstem response; OAE = otoacoustic emissions; PCHR = permanent childhood hearing loss;

UNHS = universal newborn hearing screening

**S7 Table.** Combined direct, indirect and intangible costs of hearing loss in WHO Eastern Mediterranean Region (in billion dollars)*

| Total health costs | Education costs | Productivity costs | Intangible costs | All cost |
| --- | --- | --- | --- | --- |
| $ 9.02 | $ 1.62 | $ 5.27 | $ 13.95 | $ 29.87 |

*All costs are calculated for moderate or higher degrees of hearing loss, i.e. hearing level greater than 35 dB in the better-hearing ear. The costs are estimated in 2015 International dollars (a unit of currency defined by the World Bank and represented simply as “$” in the table) (World Health Organization., 2021).

**S8 Table.** Expected impact of investing in Ear and Hearing Care (EHC) in WHO Eastern Mediterranean Region (Cost in US$)*

| Scaling up Scenario | Progressive (50%) | Ambitious (90%) |
| --- | --- | --- |
| **Total investment** | 9.4 billion | 12 billion |
| **Per capita annual investment** | .83 | .53 |
| **Number of DALYs averted** | 4.9 million | 6.1 million |
| **Monetized DALY benefits** | 28.5 million | 34.9 million |
| **Number of people benefitted** | 54.12 million | 65.13 million |
| **Productivity gains** | 37,662 million | 45,248 million |
| **Return of investment** | 6.66 | 7.02 |

* The time-frame for the analysis was set for 2020–2030. Two scenarios were considered: a ‘progress’ scenario where scale-up reaches 50% of the population by 2030; and an ‘ambitious’ scenario where scale-up addresses 90% of the population's needs by 2030 (World Health Organization., 2021).

**S1 Methods**. Database-specific search strategies

**PubMed/Medline:** ("hearing loss"[Title/Abstract] OR "deafness"[Title/Abstract] OR "hearing health"[Title/Abstract] OR "hearing aids"[Title/Abstract] OR "audiology"[Title/Abstract] OR "otolaryngology"[Title/Abstract] OR "cochlear implant"[Title/Abstract] OR "Prevalence"[Title/Abstract] OR "Otitis media"[Title/Abstract] OR "NIHL"[Title/Abstract] OR "Incidence"[Title/Abstract] OR "Hearing Screening"[Title/Abstract] OR "EHDI"[Title/Abstract] OR "UNHS"[Title/Abstract] OR "GBD"[Title/Abstract] OR "Global Burden of Hearing Impairment"[Title/Abstract] OR "Hearing Disability"[Title/Abstract] OR "Ear and Hearing Health Services"[Title/Abstract] OR "Risk of Hearing Impairment"[Title/Abstract]) AND ("education"[Title/Abstract] OR "research"[Title/Abstract] OR "early detection"[Title/Abstract]) AND ("Egypt"[Title/Abstract] OR "Iran"[Title/Abstract] OR "Iraq"[Title/Abstract] OR "Jordan"[Title/Abstract] OR "Kuwait"[Title/Abstract] OR "Lebanon"[Title/Abstract] OR "Libya"[Title/Abstract] OR "Morocco"[Title/Abstract] OR "Oman"[Title/Abstract] OR "Pakistan"[Title/Abstract] OR "Palestine"[Title/Abstract] OR "Qatar"[Title/Abstract] OR "Saudi Arabia"[Title/Abstract] OR "Somalia"[Title/Abstract] OR "Sudan"[Title/Abstract] OR "Syria"[Title/Abstract] OR "Tunisia"[Title/Abstract] OR "United Arab Emirates"[Title/Abstract] OR "Yemen"[Title/Abstract] OR "Afghanistan"[Title/Abstract] OR "Bahrain"[Title/Abstract] OR "Djibouti"[Title/Abstract] OR "Eastern Mediterranean Region"[Title/Abstract] OR "EMR countries"[Title/Abstract])

**Embase:** ('hearing loss'/exp OR 'hearing loss':ab,ti OR 'deafness'/exp OR 'deafness':ab,ti OR 'hearing aid'/exp OR 'hearing aid':ab,ti OR 'audiology'/exp OR 'audiology':ab,ti OR 'otolaryngology'/exp OR 'otolaryngology':ab,ti OR 'cochlear implant'/exp OR 'cochlear implant':ab,ti OR 'prevalence'/exp OR 'prevalence':ab,ti OR 'otitis media'/exp OR 'otitis media':ab,ti OR 'hearing screening':ab,ti OR 'hearing disability':ab,ti) AND ('education'/exp OR 'education':ab,ti OR 'research'/exp OR 'research':ab,ti OR 'early detection':ab,ti) AND ('egypt'/exp OR 'egypt':ab,ti OR 'iran'/exp OR 'iran':ab,ti OR 'iraq'/exp OR 'iraq':ab,ti OR 'jordan'/exp OR 'jordan':ab,ti OR 'kuwait'/exp OR 'kuwait':ab,ti OR 'lebanon'/exp OR 'lebanon':ab,ti OR 'libya'/exp OR 'libya':ab,ti OR 'morocco'/exp OR 'morocco':ab,ti OR 'oman'/exp OR 'oman':ab,ti OR 'pakistan'/exp OR 'pakistan':ab,ti OR 'palestine':ab,ti OR 'qatar'/exp OR 'qatar':ab,ti OR 'saudi arabia'/exp OR 'saudi arabia':ab,ti OR 'somalia':ab,ti OR 'sudan'/exp OR 'sudan':ab,ti OR 'syria':ab,ti OR 'tunisia'/exp OR 'tunisia':ab,ti OR 'united arab emirates':ab,ti OR 'yemen':ab,ti OR 'afghanistan'/exp OR 'afghanistan':ab,ti OR 'bahrain':ab,ti OR 'djibouti':ab,ti)

**Scopus:** TITLE-ABS-KEY(("hearing loss" OR "deafness" OR "hearing health" OR "hearing aids" OR "audiology" OR "otolaryngology" OR "cochlear implant" OR "prevalence" OR "otitis media" OR "hearing screening" OR "hearing disability") AND ("education" OR "research" OR "early detection") AND ("Egypt" OR "Iran" OR "Iraq" OR "Jordan" OR "Kuwait" OR "Lebanon" OR "Libya" OR "Morocco" OR "Oman" OR "Pakistan" OR "Palestine" OR "Qatar" OR "Saudi Arabia" OR "Somalia" OR "Sudan" OR "Syria" OR "Tunisia" OR "United Arab Emirates" OR "Yemen" OR "Afghanistan" OR "Bahrain" OR "Djibouti" OR "Eastern Mediterranean Region"))

**Web of Science:** TS=(("hearing loss" OR "deafness" OR "hearing health" OR "hearing aids" OR "audiology" OR "otolaryngology" OR "cochlear implant" OR "prevalence" OR "otitis media" OR "hearing screening" OR "hearing disability") AND ("education" OR "research" OR "early detection") AND ("Egypt" OR "Iran" OR "Iraq" OR "Jordan" OR "Kuwait" OR "Lebanon" OR "Libya" OR "Morocco" OR "Oman" OR "Pakistan" OR "Palestine" OR "Qatar" OR "Saudi Arabia" OR "Somalia" OR "Sudan" OR "Syria" OR "Tunisia" OR "United Arab Emirates" OR "Yemen" OR "Afghanistan" OR "Bahrain" OR "Djibouti" OR "Eastern Mediterranean Region"))

**Cochrane Library:** #1 MeSH descriptor: [Hearing Loss] explode all trees #2 MeSH descriptor: [Deafness] explode all trees
#3 MeSH descriptor: [Hearing Aids] explode all trees #4 MeSH descriptor: [Audiology] explode all trees #5 (hearing loss):ti,ab,kw OR (deafness):ti,ab,kw OR (hearing aids):ti,ab,kw OR (audiology):ti,ab,kw OR (cochlear implant):ti,ab,kw OR (otitis media):ti,ab,kw #6 #1 OR #2 OR #3 OR #4 OR #5 #7 (Egypt OR Iran OR Iraq OR Jordan OR Kuwait OR Lebanon OR Libya OR Morocco OR Oman OR Pakistan OR Palestine OR Qatar OR "Saudi Arabia" OR Somalia OR Sudan OR Syria OR Tunisia OR "United Arab Emirates" OR Yemen OR Afghanistan OR Bahrain OR Djibouti):ti,ab,kw #8 #6 AND #7

**ScienceDirect:** TITLE-ABSTR-KEY(("hearing loss" OR "deafness" OR "hearing aids" OR "audiology" OR "cochlear implant" OR "otitis media" OR "hearing screening") AND ("Egypt" OR "Iran" OR "Iraq" OR "Jordan" OR "Kuwait" OR "Lebanon" OR "Libya" OR "Morocco" OR "Oman" OR "Pakistan" OR "Palestine" OR "Qatar" OR "Saudi Arabia" OR "Somalia" OR "Sudan" OR "Syria" OR "Tunisia" OR "United Arab Emirates" OR "Yemen" OR "Afghanistan" OR "Bahrain" OR "Djibouti"))
